# Ablation of CD8α^+^ dendritic cell mediated cross-presentation does not impact atherosclerosis in hyperlipidemic mice

**DOI:** 10.1038/srep15414

**Published:** 2015-10-21

**Authors:** Bart Legein, Edith M. Janssen, Thomas L. Theelen, Marion J. Gijbels, Joep Walraven, Jared S. Klarquist, Cassandra M. Hennies, Kristiaan Wouters, Tom T.P. Seijkens, Erwin Wijnands, Judith C. Sluimer, Esther Lutgens, Martin Zenke, Kai Hildner, Erik A.L. Biessen, Lieve Temmerman

**Affiliations:** 1 Experimental Vascular Pathology, Cardiovascular Research Institute Maastricht (CARIM), University of Maastricht, The Netherlands; 2 Division of Immunobiology, Cincinnati Children’s Hospital Research Foundation, and the University of Cincinnati College of Medicine, Cincinnati, OH, United States of America; 3 Department of Internal Medicine, Cardiovascular Research Institute Maastricht (CARIM), University of Maastricht, The Netherlands; 4 Experimental Vascular Biology, Dept. of Medical Biochemistry, Academic Medical Center (AMC), University of Amsterdam, Amsterdam, The Netherlands; 5 Institute for Cardiovascular Prevention (IPEK), Ludwig Maximilians University (LMU), Munich, Germany; 6 Institute for Biomedical Engineering, Dept. of Cell Biology, RWTH Aachen University Medical School, Aachen, Germany; 7 Medical Immunology, Universitatsklinikum Erlangen, Erlangen, Germany

## Abstract

Clinical complications of atherosclerosis are almost exclusively linked to destabilization of the atherosclerotic plaque. Batf3-dependent dendritic cells specialize in cross-presentation of necrotic tissue-derived epitopes to directly activate cytolytic CD8 Tcells. The mature plaque (necrotic, containing dendritic cells and CD8 Tcells) could offer the ideal environment for cross-presentation, resulting in cytotoxic immunity and plaque destabilization. *Ldlr*^*−/−*^ mice were transplanted with *batf3*^*−/−*^ or wt bone marrow and put on a western type diet. Hematopoietic batf3 deficiency sharply decreased CD8α^+^ DC numbers in spleen and lymph nodes (>80%; P < 0,001). Concordantly, *batf3*^*−/−*^ chimeras had a 75% reduction in OT-I cross-priming capacity *in vivo*. *Batf3*^*−/−*^ chimeric mice did not show lower Tcell or other leukocyte subset numbers. Despite dampened cross-presentation capacity, *batf3*^*−/−*^ chimeras had equal atherosclerosis burden in aortic arch and root. Likewise, *batf3*^*−/−*^ chimeras and wt mice revealed no differences in parameters of plaque stability: plaque Tcell infiltration, cell death, collagen composition, and macrophage and vascular smooth muscle cell content were unchanged. These results show that CD8α^+^ DC loss in hyperlipidemic mice profoundly reduces cross-priming ability, nevertheless it does not influence lesion development. Taken together, we clearly demonstrate that CD8α^+^ DC-mediated cross-presentation does not significantly contribute to atherosclerotic plaque formation and stability.

Immune responses play a significant role in the pathophysiology of atherosclerosis^1^,^2^. They offer a promising new therapeutic angle to directly touch on pathogenic mechanisms of cardiovascular disease. Necrosis - a prime hallmark of clinical atherosclerosis - was recently linked to immunity. Necrotic tumor cell-derived epitopes are able to elicit a strong cytolitic immune response, allowing tumor elimination^3^,^4^. Key to this finding is a process called cross-presentation: direct presentation of exogenous antigen on an MHCI molecule followed by a potent CD8^+^ Tcell activation^5^. Mouse dendritic cells (CD8α^+^ or CD103^+^ DCs) appear to be highly efficient cross-presenting cells^6^, uniquely qualified to cross-present dead cell-associated antigens^7^. Identification of their human counterparts^8^,^9^,^10^,^11^,^12^ emphasizes the importance of cross-presentation in human health and disease.

In a mature atherosclerotic plaque, necrotic cell or tissue-associated epitopes, dendritic cells^13^ and CD8^+^ Tcells^14^,^15^ are abundantly present and in close contact. Significantly more DCs are found in rupture-prone, vulnerable plaques^16^, and CD8^+^ Tcells increase to up to 50% of the total leukocyte pool in human advanced plaques^17^, linking both DC and cytotoxic Tcell presence to plaque stability. In addition, CD8^+^ Tcells isolated from human plaque atherectomy specimens are highly activated, much more so than plaque CD4^+^ Tcells or Tcells isolated from the blood of the same patients^18^. Moreover, reflective of plaque-directed immunity, different auto-antigens are identified targets of immune responses in atherosclerosis. Oxidized low density lipoprotein (oxLDL) is the most well described^19^, but Tcells isolated from patients with advanced atherosclerosis also respond to F-actin, a known target in necrosis-associated cross-presentation^20^,^21^. Lastly, a recent study has demonstrated that cytotoxic CD8^+^ Tcells promote development of a vulnerable atherosclerotic plaque in mice, implicating cytolytic Tcell immunity in plaque destabilization^22^. Combining these arguments led to the following intriguing hypothesis: Cross-presentation, by mounting a cytolytic CD8^+^ Tcell immune response against cap/plaque material, might be crucial in the destabilization of the advanced plaque which generally precedes plaque rupture, thrombi formation and infarcts.

However, complete knockout of the CD8 gene in atherosclerosis-susceptible *ApoE*^*−/−*^ mice, presumably affecting both CD8α^+^ DC and CD8^+^ Tcell function, did not lead to the expected reduction in atherosclerosis^23^. Similarly, *ApoE*^*−/−*^ mice deficient in Antigen Peptide Transporter 1 (TAP1, involved in antigen cross-presentation), displayed an equivalent atherogenic response^24^. Moreover, MHCI knockout (KO) mice on a 15 week high fat diet showed increased plaque formation (+150%), suggesting that MHCI-dependent antigen presentation, inducing cytotoxic CD8^+^ Tcells, is atheroprotective^25^. Possible protection by cross-presenting DCs was also observed in the *flt3*^*−/−*^ *ldlr*^*−/−*^ mouse, where depletion of Flt3L-dependent DCs resulted in aggrevated atherosclerosis^26^. Unfortunately, each of these studies implies severe modifications of the entire immune system, which greatly impedes assessment of purely cross-presentation related effects. Thus, evidence for a direct role of cross-presentation in a “plaque-targeted” immune response remains circumstantial and inconclusive.

This study aimed at dissecting the mechanism behind the strong cytotoxic T cell response in advanced atherosclerosis. We hypothesized that cross-presentation of necrotic plaque epitopes will prime CD8^+^ Tcells to attack plaque components. In order to investigate this, we took a loss-of-function approach making use of chimeric *batf3*^*−/−*^ mice, which specifically lack CD8α^+^ DCs and CD103^+^ DCs, the most important cell populations for cross-presentation^27^,^28^. Unexpectedly, the severe defect in cross-presentation in *batf3*^*−/−*^ chimeras did not translate into apparent differences in CD8^+^ Tcell numbers, nor did it significantly affect atherosclerotic plaque size or composition.

## Results

### Cross-presentation markers increase in advanced atherosclerotic plaques

**Figure 1:**
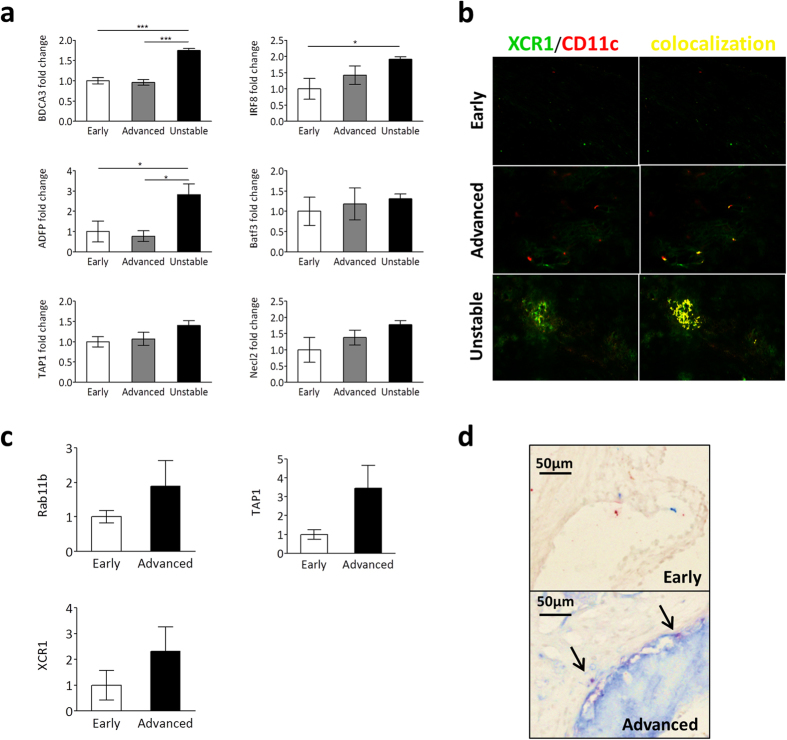
Expression of cross presentation markers in human and mouse atherosclerosis. (**a**) Total RNA was isolated from fresh-frozen human atherosclerotic plaques. Real-time PCR results of expression levels of BDCA3, IRF8, ADFP, Batf3, TAP1 and Necl2 are shown as mean ± SEM. All expression levels were first normalized for levels of β-actin expression, and are depicted as fold induction when compared to expression levels in early plaques. Samples were grouped based on histological qualification of plaque stage according to Virmani *et al*.^57^. Early: Intimal Thickening/ Pathological Intimal Thickening (n = 5), Advanced: Thick/Thin Fibrous Cap Atheroma (n = 6), Unstable: Intra Plaque Hemorrhage (n = 5). *p < 0.05, ***p < 0.001. (**b**) Representative images of frozen human carotid plaque sections (n = 8–10) doublestained with antibodies against XCR1 (green) and CD11c (red) to identify cross-presenting DCs. Colocalization was determined using a Nuance Spectral Imaging System and is indicated in yellow. (**c**) Total RNA was isolated from fresh-frozen mouse aorta’s. Real-time PCR results of expression levels of Rab11b, TAP1 and XCR1 are shown as mean ± SEM. All expression levels were first normalized for levels of GAPDH expression, and are depicted as fold induction when compared to expression levels in early plaques. Early: 8 wk old C57Bl6 mice (n = 6), Advanced: >35 wk old C57Bl6 *ApoE*^*−/−*^ mice (n = 5) (**d**) Representative images of frozen mouse aortic root sections doublestained with antibodies against CD8α (red) and CD11c (blue) to identify cross-presenting DCs. Nuclei were lightly counterstained with MethylGreen. Arrow: doublestained cell.

First, to evaluate the validity for a role of cross-presentation in plaque destabilization, expression of key cross-presentation markers in human and mouse atherosclerotic lesions was examined. We investigated RNA expression levels of Thrombomodulin, Basic leucine zipper transcription factor, ATF-like 3, Interferon regulatory factor 8 and nectin-like molecule 2 (BDCA3, Batf3, IRF8 and Necl2: markers of the main cross-presenting DC population in humans^29^) and of Antigen Peptide Transporter 1, Ras-related protein 11b, and Adipocyte Differentiation-related Protein (TAP1, Rab11b and ADFP: involved in antigen processing and presumed cross-presentation pathways^30^,^31^,^32^) in early, advanced and unstable human plaque material. BDCA3, IRF8 and ADFP were all significantly upregulated in ruptured plaques compared to initial lesions, and Batf3, TAP1 and Necl2 all showed a similar trend (Fig. 1a). Rab11b expression did not correlate with plaque progression (data not shown). XCR1^12^ and CD11c immunohistochemical staining revealed few cross-presenting cells were present in advanced and unstable human plaques, while they could not be found in early plaques (Fig. 1b, Sup. Fig. 1a). In mouse advanced plaques, Rab11b, TAP1 and XCR1 RNA expression levels were increased compared to early plaques (Fig. 1c). Similar to human plaques, cross-presenting DCs were scarce in mice and only found in advanced plaques (Fig. 1d, Sup. Fig. 1b). Overall, RNA expression patterns of cross-presentation markers correlated with a phenotype of increased plaque burden and instability, and cross-presenting cells were almost exclusively found in the more advanced plaque types, pointing to a potential role for cross-presentation in plaque progression and destabilization in human and mouse atherosclerosis.

### Cross-presentation occurs under hyperlipidemic conditions

**Figure 2:**
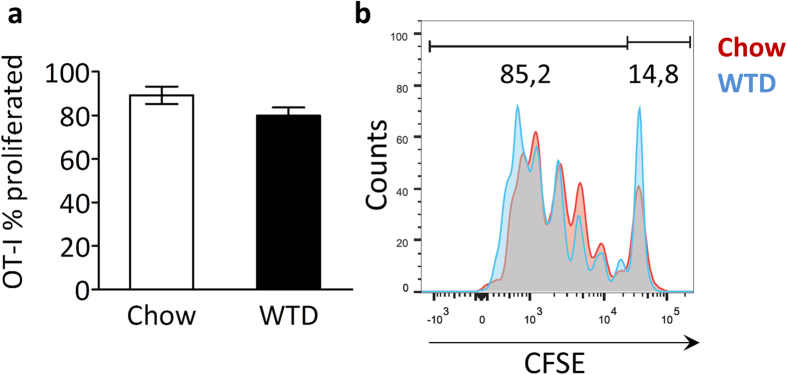
Cross-presentation occurs under hyperlipidemic conditions. *Ldlr*^*−/−*^ mice (n = 3) on a normal chow diet or fed a Western type diet (WTD) for three weeks were iv injected with irradiated OVA-expressing splenocytes and CFSE-labeled OT-I Tcells. After 72 hrs, spleens were harvested and cross-presentation was assessed by flow cytometry, quantifying the proportion of proliferating OT-I Tcells (cells with a diluted CFSE signal) within the total OT-I Tcell population, normalized for amount of injected cells. (**a**) Bar graph of proliferated OT-I Tcells (% of total OT-I Tcells) in spleen of chow or WTD-fed *ldlr*^*−/−*^ mice. (**b**) Representive CFSE dilution peaks of the OT-I Tcell population. Data are presented as mean ± SEM.

Hyperlipidemia is known to affect the behavior and activation state of many immune cell types^1^, and could thus influence the efficacy of immune responses mediated by these cells. Therefore, efficiency of cross-presentation in hyperlipidemic conditions was evaluated. *Ldlr*^*−/−*^ mice on chow or western type diet (WTD, 0.25% cholesterol) were injected with fluorescently labeled Tcells isolated from OT-I mice. These cells express a T cell receptor (TCR) engineered to recognize a specific chicken ovalbumin (OVA) antigen (SIINFEKL) only when it is presented in context of mouse MHCI-K^b^^33^. Mice also received OVA-expressing necrotic cells, which are taken up and processed by endogenous dendritic cells. Only cross-presentation of the OVA epitope leads to direct activation and proliferation of the OT-I Tcells. In chow-fed mice most OT-I Tcells had proliferated. OT-I Tcell mitogenic capacity was unaffected in WTD fed mice, establishing normal, functional cross-presentation is able to occur in a hyperlipidemic environment (Fig. 2a,b).

### Batf3-dependent DCs are efficiently depleted in atherosclerotic *batf3* ^−/−^ chimeric mice

Local inflammatory processes are very important in atherosclerosis. To ensure the effectiveness of our planned approach we tested whether vascular dendritic cells could be successfully depleted and reconstituted by a bone marrow transplant experiment. CD45.2 *ldlr*^*−/−*^ mice were lethally irradiated and received bone marrow from CD45.1 mice. Without induction of atherosclerosis, dendritic cells in the aortas of the transplanted mice were very scarce (0.8% of immune cells), and they were completely ablated 4 days after irradiation treatment (Sup. Fig. S2a, b). In addition, we could show by flow cytometry that 6 weeks after irradiation, only 1.3% of immune cells in the vessel wall are CD45.2 positive (i.e. from the host), instead they were almost exclusively CD45.1 positive, demonstrating effective reconstitution of the resident immune cells in the vessel wall by donor cells (Sup Fig S2e, f). Antibody stainings against CD45.1 and CD45.2 confirm the flow cytometry results (Sup Fig S2g). We therefore concluded that we could use a bone marrow transplantation approach to efficiently disturb cross-presentation in atherosclerosis.

**Figure 3:**
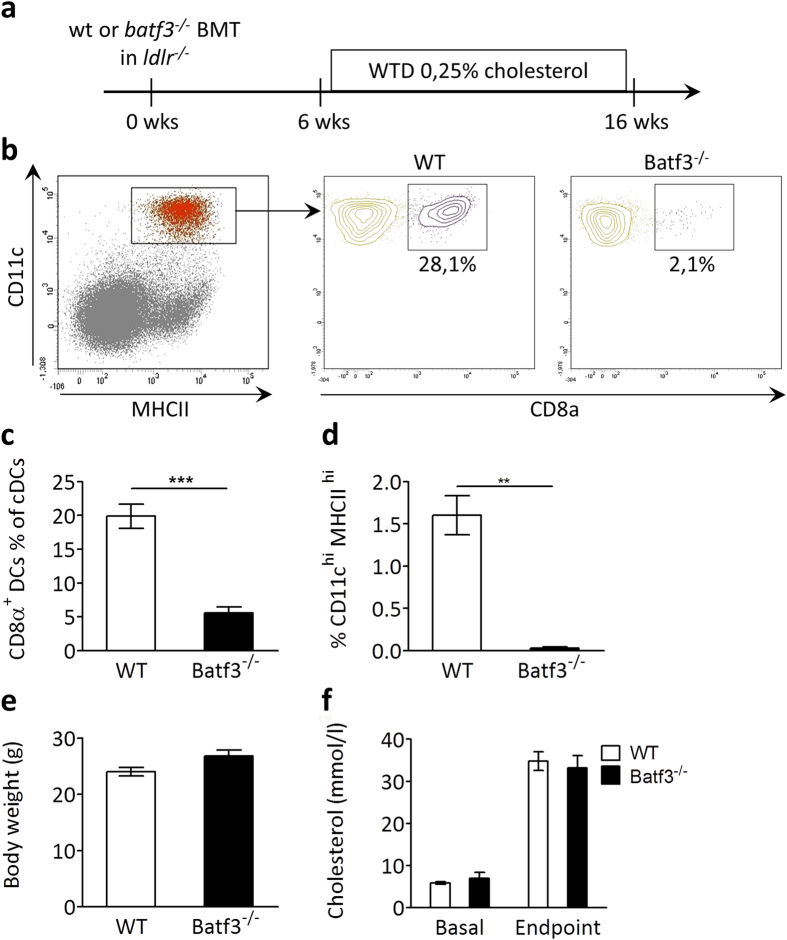
Batf3 deficiency results in severe CD8α^+^ DC depletion in the atherosclerosis model. (**a**) Lethally irradiated *ldlr*^*−/−*^ mice were reconstituted with wt (n = 15) or *batf3*^*−/−*^ (n = 12) bone marrow, and after 6 weeks recovery, put on a WTD containing 0,25% cholesterol for 10 weeks. (**b**) Representative flow cytometry gating of CD8α^+^ DC population (Lin^−^, CD11c^high^, MHCII^high^, CD8α^+^). (**c**) Bar graph of CD8α^+^ DCs as percentage of cDCs. (**d**) Bar graph of CD103^+^ DCs as percentage of cDCs. (**e**) Body weight at sacrifice. (**f**) Total cholesterol content in serum at sacrifice. Data are presented as mean ± SEM, **p < 0,01, ***p < 0,001.

In order to investigate the relative contribution of Batf3-dependent cross-presentation in development and progression of atherosclerosis, lethally irradiated *ldlr*^*−/−*^ mice were reconstituted with bone marrow from *batf3*^*−/−*^ mice or wild type (wt) control mice. *Batf3*^*−/−*^ mice selectively lack CD8α^+^ and CD103^+^ DCs and are not able to effectively cross-present necrotic cell exposed epitopes^27^. After recovery, mice were given a Western type diet (WTD) for 10 weeks to induce atherosclerotic plaque formation (Fig. 3a). *Batf3*^*−/−*^ transplanted *ldlr*^*−/−*^ mice (hereafter *batf3*^*−/−*^ chimeras) showed more than 80% reductions in CD8α^+^ DCs in spleen (Fig. 3b,c) and lymphoid organs (data not shown). As expected, CD103^+^ DCs were equally diminished by Batf3 deficiency (Fig. 3d), because their development is also Batf3 dependent^28^. Illustrating specificity of the *batf3*^*−/−*^ model, other leukocyte populations in blood (Sup. Fig. S3), spleen (Sup. Fig. S4) or peripheral lymph nodes (Sup. Fig. S5) were not affected. At sacrifice, *batf3*^*−/−*^ chimeras did not differ in body weight from mice transplanted with wt bone marrow (Fig. 3e). Both groups showed equivalent and significant increases in plasma cholesterol (Fig. 3f). These parameters indicate efficient induction of the atherosclerosis model.

We next investigated if other DC populations with, albeit lower, capacity to cross-present might have expanded to compensate for the loss of Batf3-dependent DCs. Merocytic DCs (mDCs) can cross-present in a context of diabetes^34^, and even plasmacytoid DCs (pDCs) were reported to have some cross-presentation abilities^35^. However, no differences were found in mDC or pDC numbers in spleen (Sup. Fig. S6a, b) and lymph nodes (data not shown). Recently, a subset of CD169^+^ macrophages (CD11b^+^ CD11c^+^ CD169^+^ F4/80^+^) efficiently cross-presenting tumor antigens was described in spleen^36^. This population did not change in spleens of mice on a normal diet compared to mice on a western type diet (Sup. Fig. S6c), rendering their role in atherosclerosis-related cross-presentation not very likely. In summary, we did not identify other DC or DC-like populations likely to have taken over cross-presentation from the depleted CD8α^+^ DCs in this atherosclerosis model.

### Hyperlipidemic CD8α^+^ DC depletion profoundly affects systemic cross-presentation ability

**Figure 4:**
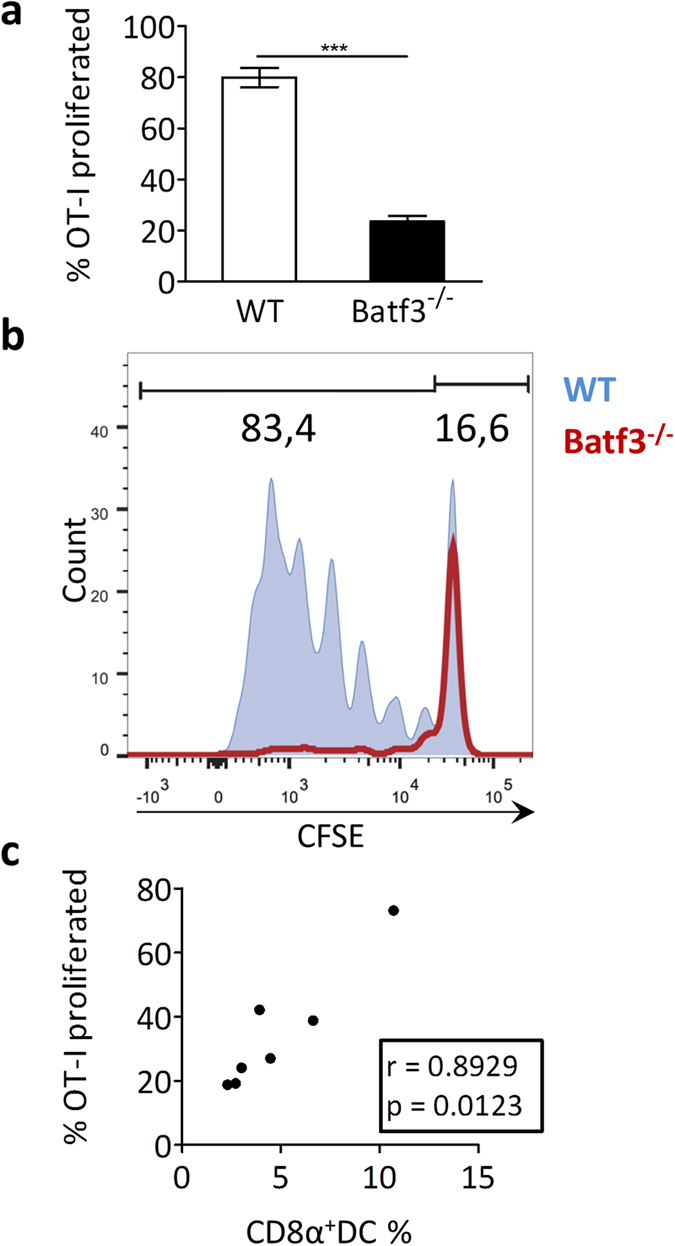
Cross-presentation is affected in *batf3*^*−/−*^ chimeric mice. *Batf3*^*−/−*^ chimeric or wt *ldlr*^*−/−*^ mice (n = 7) were iv injected with necrotic OVA-expressing splenocytes and CFSE-labeled OT-I T cells. After 72 hrs, spleens were harvested and cross-presentation was assessed by flow cytometry, quantifying the proportion of proliferating OT-I Tcells (cells with a diluted CFSE signal) within the total OT-I Tcell population, normalized for amount of injected cells. (**a**) Bar graph of proliferated OT-I Tcells (% of total OT-I Tcells) in spleen. (**b**) Representive CFSE dilution peaks of the OT-I Tcell population. (**c**) Correlation analysis between amount of residual CD8α^+^ DCs and the remaining cross-presentation capacity in *batf3*^*−/−*^ chimeras. Data are presented as mean ± SEM, ***p < 0,001.

In accordance with the severe CD8α^+^ DC depletion observed, hematopoietic Batf3 deficiency in atherosclerotic mice had a profound effect on cross-presentation. *Batf3*^*−/−*^ chimeras and control mice were injected with fluorescently labeled OT-I Tcells and with necrotic OVA-expressing cells as described above. OT-I Tcell proliferation was severely diminished from 80% in control mice to 23% in *batf3*^*−/−*^ animals (Fig. 4a,b). Interestingly, the number of residual CD8α^+^ DCs in *batf3*^*−/−*^ chimeras correlated with the cross-presenting capacity (r^2^ = 0.89, *p* = 0.01), establishing the significant role of this DC subset in cross-presentation, even in a hyperlipidemic setting (Fig. 4c).

### CD8α^+^ dendritic cell depletion does not affect atherosclerosis

**Figure 5:**
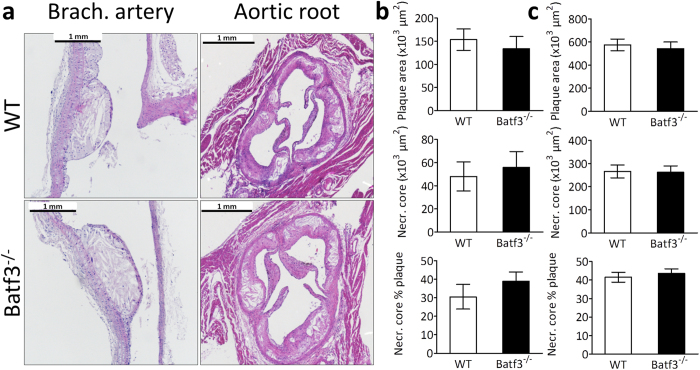
Batf3 deficiency does not influence atherosclerotic plaque size. Aortic arch and root were dissected from wt (n = 15) or *batf3*^*−/−*^ (n = 12) *ldlr*^*−/−*^ mice and analyzed by histology. (**a**) Aortic arch and root were H&E stained for plaque size analysis. (**b**, **c**) Plaque area, necrotic core area and percentage necrotic core relative to plaque area are did not differ in the brachiocephalic artey (**b**) and aortic root (**c**). Data are presented as mean ± SEM.

**Figure 6:**
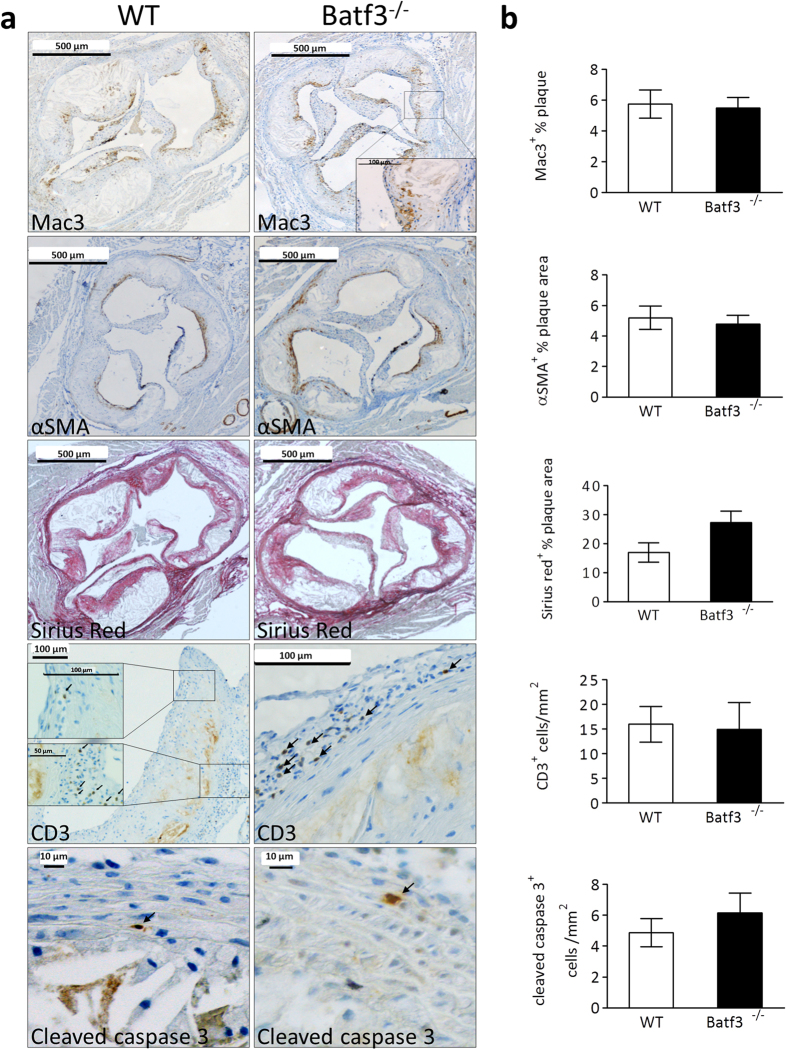
Batf3 deficiency does not influence atherosclerotic plaque composition. Aortic arch and root were dissected from wt (n = 15) or *batf3*^*−/−*^ (n = 12) *ldlr*^*−/−*^ mice and analyzed by immunohistochemistry. (**a**) Representative images of Macrophages (Mac3 staining), vascular smooth muscle cells (αSMA staining), T cells (CD3 staining), collagen (Sirius Red staining) and apoptosis (cleaved caspase 3 staining) in the aortic roots of wt and *batf3*^*−/−*^ chimeric mice. (**b**) Quantification of immunohistochemical stainings shown in (**a**). Data are presented as mean ± SEM.

First, we analyzed aortic roots from *batf3*^*−/−*^ chimeras and control mice which had been fed a normal chow diet to evaluate whether CD8α^+^ DC depletion affected initial plaque formation. However, while some mice exhibited very small initial lesions, plaque sizes of both groups were similar (Sup. Fig. 7). Next, the effect of significantly hampered cross-presentation ability on atherosclerosis could be analyzed. Unexpectedly, neither advanced plaques in the aortic root nor initial plaques in brachiocephalic artery showed differences in plaque size, necrotic core size or necrotic core percentage between *batf3*^*−/−*^ chimeras and control mice (Fig. 5a,b). Plaques from *batf3*^*−/−*^ chimeras and control mice also contained the same amount of macrophages (Fig. 6a,b: first panel). In addition, features of plaque stability were similar in both groups, as we observed no changes in vascular smooth muscle cell content or collagen (Fig. 6a,b: second and third panel, Sup. Fig. S9). To exclude unknown local environmental or other contributory factors, we repeated the study in the same setup in the laboratory of our collaborator Prof. Dr. E. Janssen, Cincinnati, US, with *ldlr*^*−/−*^ and *batf3*^*−/−*^ mice from Jackson Laboratories. Again, cross-presenting CD8α^+^ DCs were severely depleted in *batf3*^*−/−*^ chimeras, yet no differences were seen in atherosclerosis phenotype (Sup. Fig. S8). Thus, CD8α^+^ DC depletion does not alter plaque size or the stable plaque phenotype in atherosclerotic mice.

### Tcell activation is unchanged in CD8α^+^ DC depleted atherosclerotic mice

**Figure 7:**
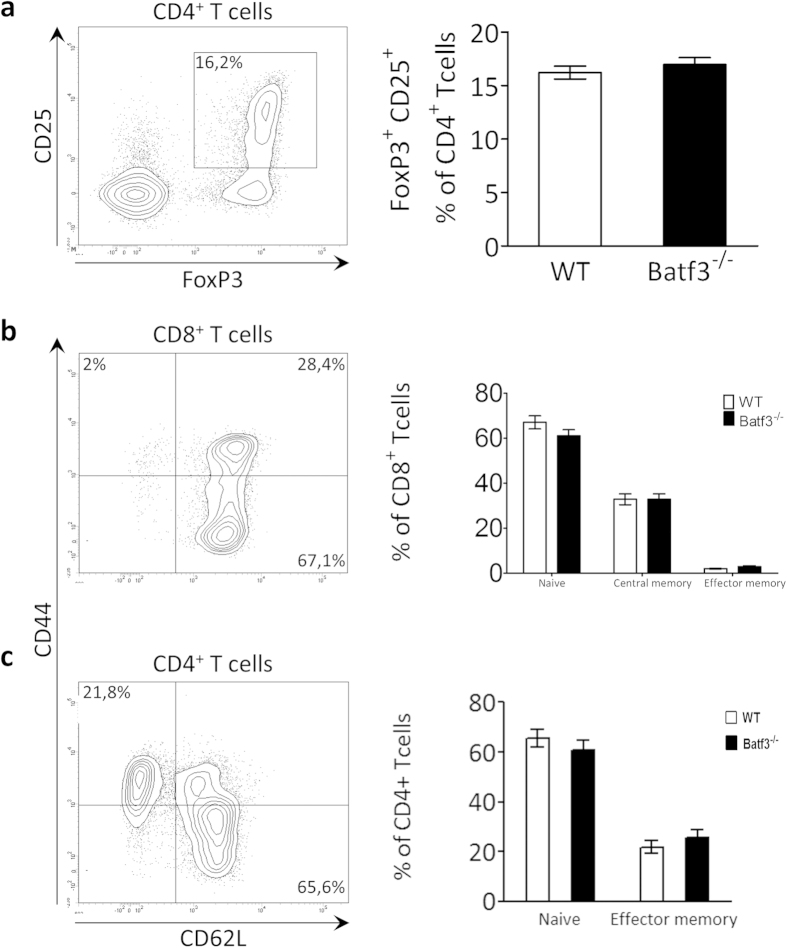
Tcell numbers are unchanged in *batf3*^*−/−*^ chimeras. Tcell subset numbers were analyzed in the aorta-draining lymph node by flow cytometry. (**a**) CD25^+^, FoxP3^+^ regulatory Tcells cell are presented relative to the CD4^+^ Tcell population. (**b**) Naïve (CD62L^hi^, CD44^lo^), central memory (CD62L^hi^, CD44^hi^) and effector memory (CD62L^lo^, CD44^hi^) populations are presented as percentages of CD8^+^ Tcells. (**c**) Naïve (CD62L^hi^, CD44^lo^), and effector memory (CD62L^lo^, CD44^hi^) populations are presented as percentages of CD4^+^ Tcells. Data are presented as mean ± SEM.

We postulated that cross-presentation of plaque epitopes would lead to expansion of cytolytic plaque-targeted CD8^+^ Tcells, resulting in plaque destabilization. However, consistent with the observations regarding plaque size or phenotype, Tcell content and plaque apoptosis did not differ between *batf3*^*−/−*^ chimeric mice and control mice (Fig. 6a,b: fourth and fifth panel). Moreover, total, CD4^+^ and CD8^+^ Tcell numbers in blood, spleen and peripheral lymph nodes and were not changed by *batf3* deficiency (Sup. Fig S3-5). As we would primarily expect effects on T cell biology at the site of atherosclerosis, we also analyzed T cell phenotype in the aorta-draining lymph nodes (lnn. mediastinalis dorsalis, strongly enlarged in atherosclerosis) but no relevant differences in the proportion of regulatory T cells (Fig. 7a) were found. Naïve (CD44^low^, CD62L^high^), effector memory (CD44^high^,CD62L^low^) and central memory Tcell counts (CD44^high^, CD62L^high^) in the aorta-draining lymph nodes were not affected by Batf3 deficiency (Fig. 7b,c) as well. These data suggest that cross-presentation does not play an active role in the clonal expansion of atherosclerosis-relevant Tcells, neither locally in the aorta-draining lymph node, or systemically in the lymphoid organs.

## Discussion

Cytotoxic immunity is emerging as a key process in advanced atherosclerosis^22^, but its actors and triggers are hitherto largely unknown. We opted for cross-presentation as plausible candidate, considering that all components for effective cross-presentation are present in the advanced atherosclerotic plaque and that several genes involved in cross-presentation were more expressed in ruptured compared to early atherosclerotic lesions of CVD patients. Moreover, exposure to high LDL/VLDL levels in advanced atherosclerosis would most likely not interfere with the cross-presentation machinery, as we showed that systemic cross-presentation efficacy in mice was not affected by hyperlipidemia. Likewise, CD11c^+^ DCs under conditions of hyperlipidemia take up and process antigens normally, and are able to activate Tcells^37^.

Cross-presentation of necrotic plaque epitopes could theoretically take place in the plaque itself, in analogy to antigen presentation by DCs to CD4^+^ T cells^38^, or in plaque-draining lymphoid organs. CD103^+^ DCs increase in the atherosclerotic aortic wall^26^ and might activate CD8^+^ T cells *in situ* or migrate to adjacent lymph nodes. Alternatively, CD8α^+^ DCs could cross-present shed plaque material in lymphoid organs, as they very efficiently do so with dying cell particles during intracellular pathogen infections^39^, upon which activated CD8^+^ T cell clones may travel to the plaque. Here, both routes of cross-presentation were ablated by depleting CD8α^+^ DC and CD103^+^ DC in a well-established mouse model of atherosclerosis. Concordant with previous studies in whole-body *batf3*^*−/−*^ mice^27^,^28^, chimeric *batf3*^*−/−*^ mice exclusively targeted the aforementioned Batf3 dependent cell populations, leaving other leukocyte subsets unaffected. In addition, cross-presentation capability – again similar to the full *batf3*^*−/−*^ phenotype – was profoundly reduced in *batf3*^*−/−*^ chimeras with a more than 70% loss of OVA-OT-I cross priming capacity. Moreover, a strong correlation between the amount of residual CD8α^+^ DCs and the ability to cross-present OVA to OT-I Tcells could be established. CD8α^+^ DCs can develop independently of Batf3 and in conditions of infection compensatory *batf3*^*−/−*^ CD8α^+^ DC development was reported^40^,^41^. Nevertheless, effective numerical as well as functional depletion of this subset suggests that any batf3-independent CD8α^+^ DC development is not opportune for the present study setup.

Remarkably, the severe CD8α^+^ and/or CD103^+^ DC cross-presentation defect did not alter atherosclerotic plaque phenotype in *batf3*^*−/−*^ chimeric mice. This is in agreement with the reported lack of effect of TAP1 deficiency, which transports antigen-MHCI complexes to the cell surface, on plaque formation in *ApoE*^*−/−*^ mice^24^, albeit that the interpretation of this study was complicated by reductions in peripheral CD8^+^ Tcell numbers^42^. By contrast, MHCI KO mice develop 150% bigger plaques when fed a high fat diet for 15 weeks^25^. However, apart from being unable to cross-present, MHCI deficiency influences a broad range of stromal and hematopoietic cells. These mice suffer from CD8^+^ lymphocytopenia, and profound iron overload^43^, which can both impact atherosclerosis development^22^,^44^. Similarly, loss of function studies in *flt3*^*−/−*^ *ldlr*^*−/−*^ mice suggested an athero-protective role of aortic CD103^+^ DCs, possibly by increasing regulatory Tcells in the lesion^26^. Of note, Flt3 is involved in the development of several types of hematopoietic cells^45^, and its deficiency affects Tcells and several DC subsets systemically and directly as well^46^. Our study setup differs from the above-mentioned studies in the fact that we achieve specific functional targeting of cross-presenting cell populations, allowing us to evaluate for the first time their single contribution to atherosclerosis development.

Even so, cross-presentation of necrotic plaque epitopes could be mediated by other cell populations, which were not targeted with the *batf3*^*−/−*^ model. Therefore, subsets with reported cross-presentation ability such as mDCs^34^, pDCs^35^ or CD169^+^ macrophages^36^ were analyzed. PDCs are present in scarce amounts in the intima of atherosclerotic arteries, but their role in atherosclerosis remains inconclusive^47^,^48^. The role of mDCs or CD169^+^ macrophages in CVD is hitherto unknown. Investigating cross-presentation of plaque epitopes by those cell types would require a specific mDC knockout model (not available to date) or combining the inducible CD169-DTR macrophage knockout model^49^ with an atherosclerosis model. Nevertheless, we did not find any relevant expansion of these populations in *batf3*^*−/−*^ chimeras, rendering a compensatory effect in Batf3 deficiency unlikely.

We postulated that cross-presentation deficiency would reduce atherosclerosis by failing to induce cytotoxic CD8^+^ Tcells involved in plaque vulnerability^22^. However, in accordance with the unchanged plaque phenotype, Tcell subset numbers in blood and lymphoid organs as well as in plaques of chimeric *batf3*^*−/−*^ mice were similar to those in wt controls. This suggests that CD8α^+^ and CD103^+^ DCs cannot account for the marked increase in CD8^+^ Tcells in advanced atherosclerotic plaques^17^. In analogy to Cytomegalovirus infection, where priming of CD8^+^ Tcells is largely dependent on Batf3-cross-presentation only in disease onset and not during latent infection^50^, cross-presentation by Batf3-dependent cells in the chronic stages of advanced atherosclerosis could be obsolete. In support of this view, it has been reported that only apoptotic cells (much more abundant in initial atherosclerotic lesions) elicit mature functional CD8^+^ Tcells^51^. Necrotic cells, which hallmark advanced atherosclerosis, may well fail to induce sufficient CD40 expression on DCs, which is an essential step to subsequent CD8^+^ Tcell activation. Alternatively, it has been shown that apoptotic tissue antigens are cross-presented to tolerize autoreactive CD8^+^ Tcells^52^ and that sustained cross-priming by CD8α^+^ DCs can result in tolerance^53^. Vaccination studies using tolerogenic DCs loaded with oxLDL or ApoB100 have a positive effect on atherosclerotic disease progression^54^,^55^. However, as severe CD8α^+^ DC depletion did not increase plaque burden, a cross-tolerogenic role for CD8α^+^ DCs in atherosclerosis seems unlikely.

In summary, Batf3 deficiency in hyperlipidemic conditions leads to a highly specific, severe defect in cross-presentation, with no effect on Tcell immunity or other leukocyte subsets. We clearly demonstrate that CD8α^+^/CD103^+^ DC-dependent cross-presentation does not impact atherosclerotic plaque size or features of plaque stability and consequently has no major causal role in plaque rupture or the generation of a cardiovascular event. Taken together, we present convincing evidence that the contribution of cross-presentation of atherogenic antigens to atherosclerotic plaque progression is marginal at best. Our study thereby raises the intriguing possibility that in advanced atherosclerosis CD8^+^ Tcell immunity is steered by other mechanisms, involving for instance Th1 Tcell activation^56^, which warrants further efforts to dissect the driving forces in cytolytic plaque-attacking Tcell generation.

## Methods

### RNA isolation from human atherosclerotic plaque lesions

Total RNA was extracted from freshly frozen atherosclerotic tissue samples obtained from endarterectomy surgery. Collection, storage in the Maastricht Pathology Tissue Collection (MPTC) and patient data confidentiality as well as tissue usage were in accordance with the “Code for Proper Secondary Use of Human Tissue in the Netherlands” (http://www.fmwv.nl, http://www.federa.org/sites/default/files/digital_version_first_part_code_of_conduct_in_uk_2011_12092012.pdf). Tissue samples destined for RNA isolation were snap-frozen immediately after resection, staged by histological analysis of adjacent tissue sections according to Virmani *et al*.^57^ and grouped as early lesions (IT: intimal thickening/PIT: pathological intimal thickening, n = 5), advanced lesions (Tk/Tn FCA: thick or thin fibrous cap atheroma, n = 6) or advanced unstable lesions (IPH: intra plaque hemorrhage, n = 5). RNA was isolated with the Guanidine Thiocyanate (GTC)/CsCl gradient method and the NucleoSpin RNA II kit (Macherey-Nagel GmbH & Co. KG)^58^. RNA concentration was determined using the Nanodrop ND-1000 (Thermo Scientific) and quality was assessed by RNA 6000 Nano/Pico LabChip (Agilent 2100 Bioanalyzer, Palo Alto, CA, USA) analysis based on RIN (RNA integration number) values. RIN values above 5.6 were considered acceptable.

### RNA isolation from mouse aorta

Total RNA was extracted from freshly frozen mouse aorta. For early plaques 6 8 weeks old C57BL/6 mice were used, for advanced plaques 5 C57BL6 *ApoE*^*−/−*^ mice of over 35 weeks old were used. Snap-frozen aorta was disrupted using Trizol (Life Technologies), glass beads and a Mini-Beadbeater. RNA isolation was then performed using the Qiagen RNAeasy Micro Kit following manufacturer’s instructions. RNA concentration and purity was determined on a Nanodrop 2000 spectrophotometer.

### Real-time PCR on human and mouse atherosclerotic plaque lesions

500 ng total plaque RNA was cDNA transcribed with the iScript cDNA Synthesis Kit (BioRad) following manufacturer’s instructions. Real time PCR was performed for expression of human TAP1, ADFP, BDCA3, IRF8, Rab11b, Necl2 and Batf3 or mouse Rab11b, TAP1 and XCR1 using SensiMix SYBR Green (Bio-Rad) on a Bio-Rad CFX96 Real-Time System, C1000 Thermal Cycler. Gene expression of one housekeeping gene, i.e. human β-actin or mouse GAPDH, was assessed for normalization. Due to the limited quantity of plaque material, more house-keeping genes could not be included in the analysis. Nevertheless, for analysis of plaque material human β-actin and mouse GAPDH are both considered stable housekeeping genes within our laboratory, based on various qPCR experiments to select a viable housekeeping gene for atherosclerotic plaques (data not shown). Gene specific intron-spanning primers (Eurogentec) were designed with Roche Applied Science’s Universal ProbeLibrary Assay Design Center (Supplemental Table I). For validation of primer specificity a primer BLAST (NCBI) specificity analysis was performed. Real time PCR data was analyzed using Bio-Rad CFX Manager v2.0 Software.

### Immunohistochemistry and colocalization on human plaque sections

The co-localization of the a DC marker with a marker for cross-presentation in human plaques was measured by multispectral imaging of immunohistochemical staining. Frozen human plaque sections were stained for CD11c (BD Pharmingen) and XCR1 (Novus Biologicals). From double staining, spectral imaging data sets from maximal three random regions of interest were taken between 420–720 nm (10 nm interval) at a 5× as well as at a 20× magnification using a Nuance spectral imaging system (Perkin Elmer/Caliper Life Sciences, Hopkinton, MA, USA) mounted on a Zeiss Axiophot microscope. Slides stained for a single chromogen (Vector Red and Vector Blue, both Vector Laboratories) only were used to create a spectral library. The spectral library was used for computational segregation of the individual image components using the NuanceTM 3.0.2 software as described^59^. After spectral unmixing, pseudo-colors were assigned to unmixed images, and composite images showing co-localization were generated with the Nuance 3.0.2 software.

### Animals

All animal work was approved by the local regulatory authority of Maastricht University and in accordance with EU and Dutch government laws and guidelines. Mouse experiments performed in Cincinnati (US) complied with approved Institutional Animal Care and Use Committee guidelines and the guidelines of the Association for Assessment and Accreditation of Laboratory Animal Care International. Male *ldlr*^*−/−*^ mice were obtained from the Jackson Laboratory (Bar Harbor, ME) and had been backcrossed at least 10 generations on a C57BL/6J background. For CD45.1/2 studies male *ldlr*^*−/−*^ mice have been crossed in-house at our SPF breeding facility into the CD45.1 background. *Batf3*^*−/−*^ mice were a kind gift from Prof. Dr. K. Hildner (Uniklinikum Erlangen, Germany) or purchased directly from the Jackson Laboratory. OT-I mice were a gift from Prof. Dr. M. Zenke (Uniklinikum Aachen, Germany) or purchased at the Jackson Laboratory and crossed to the CD45.1 (B6.SJL-*Ptprca Pepcb*/BoyJ) background at the Cincinnati in-house SPF mouse breeding facility. B6. PL-*Thy-1a*/Cy (CD90.1) mice and C3H Act-mOVA mice were bred in the Cincinnati in-house SPF mouse breeding facility. All mice were fed a standard diet (Cat# V1535, sniff Spezialdiäten GmbH, Soest, Germany) unless indicated otherwise, had *ad libitum* access to food and water and were housed under a 12 hour light-dark cycle.

### Bone marrow transplantation and atherosclerosis induction in mice

Male C57BL/6 CD45.2 *ldlr*^*−/−*^ mice of at least 12 weeks of age were housed under filter top cages and given antibiotics supplemented water (Neomycin (100 mg/L; Gibco, Carlsbad, CA, USA) and Polymyxin B sulfate (60.000 U/L; Gibco)), starting 2 weeks before until 6 weeks after bone marrow transplantation. To induce bone marrow aplasia, *ldlr*^*−/−*^ mice (n = 69) were exposed to two doses of 6 Gy total body irradiation (0.5 Gy/min, Philips MU15F/225kV, Hamburg, Germany) one day before bone marrow transplantation, with 12 hrs recuperation time in between each dose. Irradiated recipients (Maastricht study n = 15 wt, n = 12 *batf3*^*−/−*^, Cincinnati study n = 15 for both groups, CD45.1/2 study n = 12) were injected via tail vein with bone marrow cell suspensions (10^6^ cells/mouse), prepared from homozygous C57BL/6J *batf3*^*−/−*^ female donor mice or wt littermate controls by tibia/ femur lavage. For the CD45.1/2 study, donor mice were male C57BL/6 CD45.1 *ldlr*^*−/−*^. For atherosclerosis induction, mice were allowed to recover for 6 weeks after bone marrow transplantation, blood samples were taken from the tail vein and mice were put on a Western type diet (WTD) containing 0,25% cholesterol (Special Diets Services, Witham, Essex, UK) for 10 weeks. At sacrifice, mice were euthanized by a pentobarbital overdose (115 mg/kg), injected intraperitoneally. Blood was taken by left ventricular puncture. Spleen, aortic lymph nodes and a mix of peripheral lymph nodes (axillary, mesenteric, mandibular, aorta-draining lymph nodes (lnn. mediastinalis dorsalis, located in the precordial mediastinum: a group of two to four larger dorsal nodes attached to the thymus cranial to the aortic arch and lateral to the cranial caval veins) ) were isolated. For flow cytometry experiments, aorta and carotids were dissected before perfusion. For histological sampling, mice were perfused with phosphate buffered saline (PBS) (NaCl/Na_2_HPO_4_/KH_2_PO_4_, pH 7.4) containing sodium nitroprusside (0.1 mg/ml, Sigma) and 1% paraformaldehyde (PFA) and heart, aorta and carotids were dissected.

### Histology and immunohistochemistry of mouse atherosclerotic lesions

After isolation, the carotid arteries, aorta and the heart were fixed overnight in 1% PFA and paraffinembedded sections (4 μm) were cut. For frozen sections, aortic root was snap-frozen in OCT, and 4 μm frozen sections were cut. To determine plaque volume and necrotic core content in the aortic arch and aortic root, plaque area and necrotic core were measured on four consecutive H&E stained sections at 20 μm intervals that covered the entire lesion and averaged, as described before^60^ . In the aortic root, measurements were calculated for each valve separately and then added to obtain total root plaque area and necrotic core size.

Collagen content was detected by Sirius Red (Sigma) staining and expressed as a percentage of plaque area. Slides were analyzed in a blinded manner using a Leica DM3000 light microscope (Leica Microsystems, Wetzlar, Germany) coupled to a computerized morphometric system (Leica Qwin 3.5.1). Immunohistochemical stainings were performed on paraffin or frozen aortic root sections for CD3 (DAKO, Glostrup, Denmark), α‐smooth muscle actin (ASMA) (DAKO), Mac3 (BD), cleaved caspase 3 (Cell Signaling), CD11c (supernatant of N418 Hybridoma Cells), CD8α (Thermo Scientific), biotinylated CD45.1 (BD Biosciences) or biotinylated CD45.2 (BD Biosciences). Slides were analyzed blindly using a Leica Qwin program (for ASMA and Mac3) or counted manually (for CD3 and cleaved caspase 3). The amount of positive cells was expressed as percentage positively stained area per total plaque area (for ASMA and Mac3) or as number of positive cells per mm^2^ plaque area (for CD3 and cleaved caspase 3).

### Plasma cholesterol analysis

Cholesterol levels in plasma were measured in duplicate using a colorimetric assay (DiaSys, Diagnostic Systems) according to the kit’s instructions.

### Flow cytometry

Blood, spleen, aortic lymph nodes and peripheral lymph nodes (a mixture of mesenteric, mandibular and axillary lymph nodes) were removed before perfusion, gently dissociated through a 70 μm cell strainer (Greiner), treated with erylysis buffer (8.4 g NH_4_Cl, 0.84 g NaHCO_3_ in 1l PBS) and stained for total leukocytes (CD45^+^, BioLegend), total T cells (CD3^+^, eBioscience), T helper cells (CD4^+^, BD Bioscience), cytotoxic T cells (CD8α^+^, BD Bioscience), B cells (B220^+^, BD Bioscience), NK cells (CD3^−^ NK1.1^+^, BD Bioscience) monocytes (CD11b^high^ Ly6G^low^, BD Bioscience), granulocytes (CD11b^high^ Ly6G^high^, BD Bioscience), conventional dendritic cells (cDCs; CD11c^high^ MHCII^high^, either CD8^−^ CD11b^+^, double negative CD8^−^ CD11b^−^ or CD8^+^/CD103^+^ CD11b^−^, eBioscience) and plasmacytoid DCs (pDCs; PDCA‐1^high^ B220^+^, eBioscience). T cell subtypes were analyzed performing additional cell surface staining on FoxP3 (eBioscience), CD44 (BD Bioscience) and CD62L (eBioscience). Cross presenting macrophages were analyzed using a cocktail of CD45 (BioLegend), CD3 (eBioscience), CD19 (eBioscience), CD11c (eBioscience), CD11b (BD Bioscience), F4/80 (BioLegend), and CD169 (BioLegend), and defined as CD45^+^ CD3/CD19^−^ CD11c^−^ CD11b^+^ F4/80^+^ CD169^+^. For cDC and pDC analysis, spleen and lymph nodes were pretreated for 30 minutes with a cocktail of liberase (32 μg/ml, Roche) and DNase (0.8 μg/ml, Roche) in RPMI medium (Gibco). Absolute cell numbers in blood were calculated by use of Trucount tubes (BD Bioscience). All flow cytometry analysis was performed on a BDCanto II (BD Bioscience) using FACS Diva Analysis Software vs6.

### Flow cytometry of mouse aorta

Aortic arch, carotids and thoracic aorta were dissected, transferred to an enzymatic cocktail consisting of hyaluronidase (85 U/ml, Sigma), liberase (32 μg/ml, Roche) and DNase (0.8 μg/ml, Roche) in RPMI medium (Gibco) and with forceps and syringe dissociated in pieces small enough to be taken up with a 1 ml Greiner pipet. Tissue was incubated in this enzymatic cocktail for 1 hour at 37 degrees with regular shaking and filtered through a 70 μm cell strainer (Greiner). Two aortas were pooled together for consequent FACS analysis and samples were stained with a cocktail of CD45 (BioLegend), CD3 (eBioscience), CD19 (eBioscience), NK1.1 (eBioscience), Ly6G (eBioscience), F4/80 (eBioscience), CD11c (eBioscience), MHCII (eBioscience), CD45.1 (BD Biosciences) and CD45.2 (BD Biosciences). CD3, CD19, Ly6G and F4/80 were used as dumb gate to identify CD45^+^CD11c^high^, MHCII^high^ dendritic cells. Analysis was performed on a BDCanto II (BD Bioscience) using FACS Diva Analysis Software vs6.

### OT – I cross presentation analysis

*Batf3*^*−/−*^ or wt *ldlr*^−/−^ recipient mice (n = 3– 8) on chow or high fat diet received intravenous 5 × 10^4^ CFSE‐labeled (Life Technologies) purified OVA specific OT‐I/CD45.1 CD8^+^ T cells together with 5 × 10^5^ purified CD90.1 wt CD8^+^ T cells that served as an internal control. All injected CD8^+^ T cells were purified using the CD8^+^ T Cell Isolation Kit II (Miltenyi Biotec GmbH, Bergisch Gladbach, Germany) according to the kit’s manual. The next day, mice received i.v. 5 × 10^5^ irradiated (1500 rad) C3H‐actmOVA splenocytes. Three days later, spleen and lymph nodes were isolated and stained for CD8 (BioLegend), Vα2 (BioLegend), CD45.1 (BD) and CD90.1 (BioLegend). Subsequently, OT‐I/CD90.1 proliferation and expansion were determined based on CFSE dilution and the ratio of OT‐I/CD45.1 to CD90.1 control CD8^+^ T cells.

### Statistics

All data is presented as mean ± SEM. Data was processed using GraphPad Prism 5 (Graph Pad Software Inc., San Diego, CA, USA). Individual groups of normally distributed data were analyzed with a Student’s *t*-test, otherwise a non-parametric Mann-Whitney *U* test was used. Data containing more than two groups was analyzed with 1-way ANOVA or the non-parametric Kruksal-Wallis test, and results were corrected for multiple testing. Correlation analysis was performed using a Spearman correlation test. Different outcomes were considered significant on several levels: *p < 0.05, **p < 0.01, ***p < 0.001.

## Additional Information

**How to cite this article**: Legein, B. *et al*. Ablation of CD8α^+^ dendritic cell mediated cross-presentation does not impact atherosclerosis in hyperlipidemic mice. *Sci. Rep.* **5**, 15414; doi: 10.1038/srep15414 (2015).

## Supplementary Material

Supplementary Information
