# Author Correction: Ablation of CD8α^+^ dendritic cell mediated cross-presentation does not impact atherosclerosis in hyperlipidemic mice

**DOI:** 10.1038/s41598-020-65653-2

**Published:** 2020-05-26

**Authors:** Bart Legein, Edith M. Janssen, Thomas L. Theelen, Marion J. Gijbels, Joep Walraven, Jared S. Klarquist, Cassandra M. Hennies, Kristiaan Wouters, Tom T. P. Seijkens, Erwin Wijnands, Judith C. Sluimer, Esther Lutgens, Martin Zenke, Kai Hildner, Erik A. L. Biessen, Lieve Temmerman

**Affiliations:** 1 Experimental Vascular Pathology, Cardiovascular Research Institute Maastricht (CARIM), University of Maastricht, Maastricht, The Netherlands; 2 Division of Immunobiology, Cincinnati Children’s Hospital Research Foundation and the University of Cincinnati College of Medicine, Cincinnati, OH United States of America; 3 Department of Internal Medicine, Cardiovascular Research Institute Maastricht (CARIM), University of Maastricht, Maastricht, The Netherlands; 4 Experimental Vascular Biology, Dept. of Medical Biochemistry, Academic Medical Center (AMC), University of Amsterdam, Amsterdam, The Netherlands; 5 Institute for Cardiovascular Prevention (IPEK), Ludwig Maximilians University (LMU), Munich, Germany; 6 Institute for Biomedical Engineering, Dept. of Cell Biology, RWTH Aachen University Medical School, Aachen, Germany; 7 Medical Immunology, Universitatsklinikum Erlangen, Erlangen, Germany

Correction to: *Scientific Reports* 10.1038/srep15414, published online 21 October 2015

This Article contains an incorrect version of the Supplementary Information file. The correct Supplementary Information file has been provided below.

## Supplementary information

Supplementary Information.

