# Supplementary material for: Ablation of CD8α+ dendritic cell mediated cross-presentation does not impact atherosclerosis in hyperlipidemic mice
Source: Sci Rep. 2015 Oct 21;5:15414. doi: 10.1038/srep15414 (PMC4614009; doi:10.1038/srep15414)
Supplement: Supplementary Information [file srep15414-s1.pdf]

Supplemental Material

Ablation of CD8α$^+$ dendritic cell mediated cross-presentation does not impact atherosclerosis in hyperlipidemic mice

Bart Legein[1], Edith Janssen[2], Marion Gijbels[1], Joep Walraven[1], Jared Klarquist[2], Cassandra Hennies[2], Thomas Theelen[1], Tom T.P. Seijkens[3], Erwin Wijnands[1], Esther Lutgens[3,4], Martin Zenke[5], Kai Hildner[6], Erik A.L. Biessen[1], *Lieve Temmerman[1]

[1]Experimental Vascular Pathology, Cardiovascular Research Institute Maastricht (CARIM), University of Maastricht, The Netherlands

[2]Division of Immunobiology, Cincinnati Children's Hospital Research Foundation, and the University of Cincinnati College of Medicine, Cincinnati, OH, United States of America

[3]Experimental Vascular Biology, Dept. of Medical Biochemistry, Academic Medical Center (AMC), University of Amsterdam, Amsterdam, The Netherlands

[4]Institute for Cardiovascular Prevention (IPEK), Ludwig Maximilians University (LMU), Munich, Germany

[5]Institute for Biomedical Engineering, Dept. of Cell Biology, RWTH Aachen University Medical School, Aachen, Germany

[6]Medical Immunology, Universitatsklinikum Erlangen, Erlangen, Germany

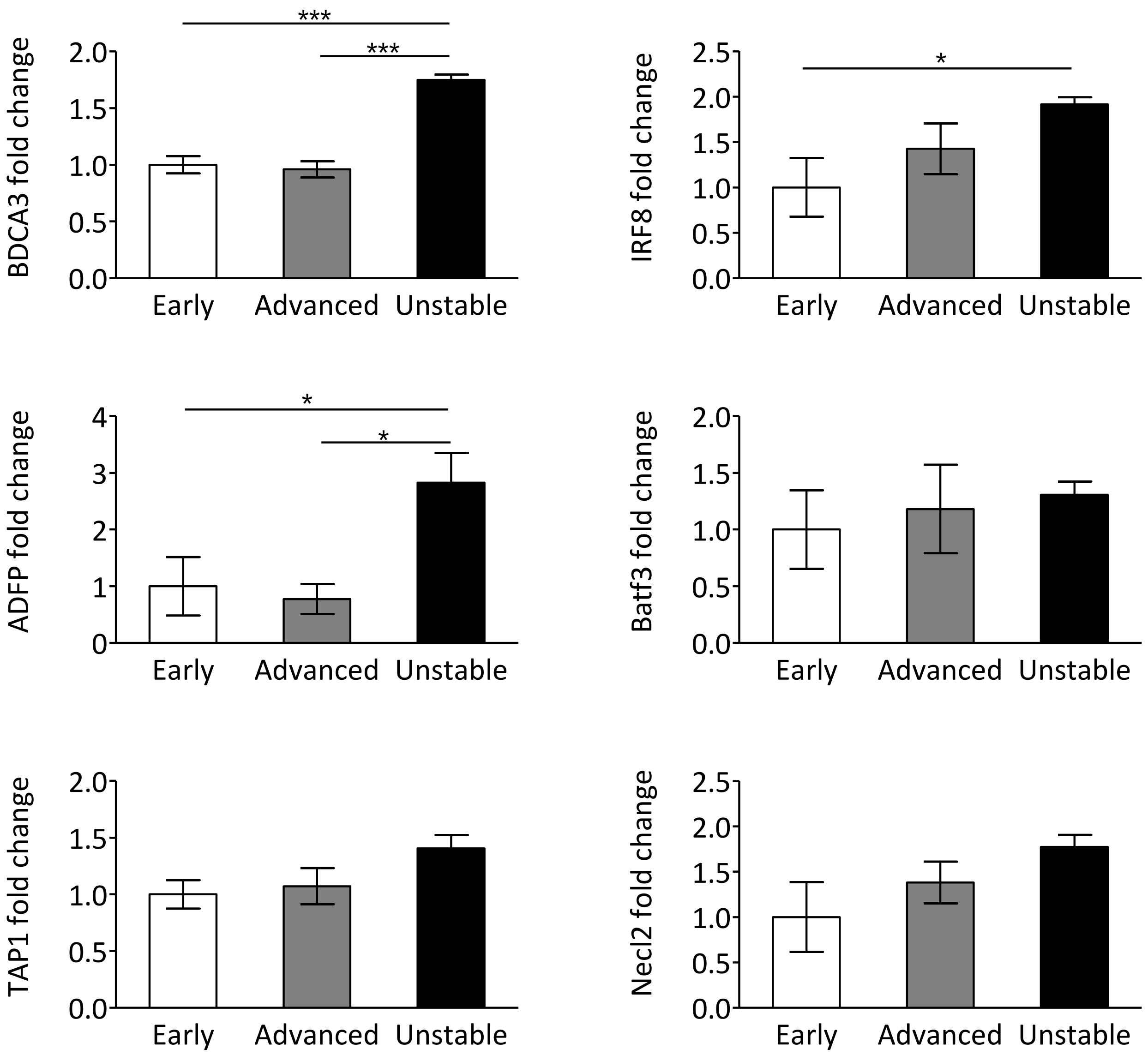

Supplementary Figure S1. Expression of cross presentation markers in human atherosclerosis

Total RNA was isolated from fresh-frozen human atherosclerotic plaques. Real-time PCR results of expression levels of BDCA3, IRF8, ADFP, Batf3, TAP1 and Necl2 are shown as mean SEM. All expression levels were first normalized for levels of β-actin expression, and are depicted as fold induction when compared to expression levels in early plaques. Samples were grouped based on histological qualification of plaque stage according to Virmani *et al.* [57]. Early: Intimal Thickening/ Pathological Intimal Thickening (n=5), Advanced: Thick/Thin Fibrous Cap Atheroma (n=6), Unstable: Intra Plaque Hemorrhage (n=5). *: $p<0.05$, ***: $p<0.001$.

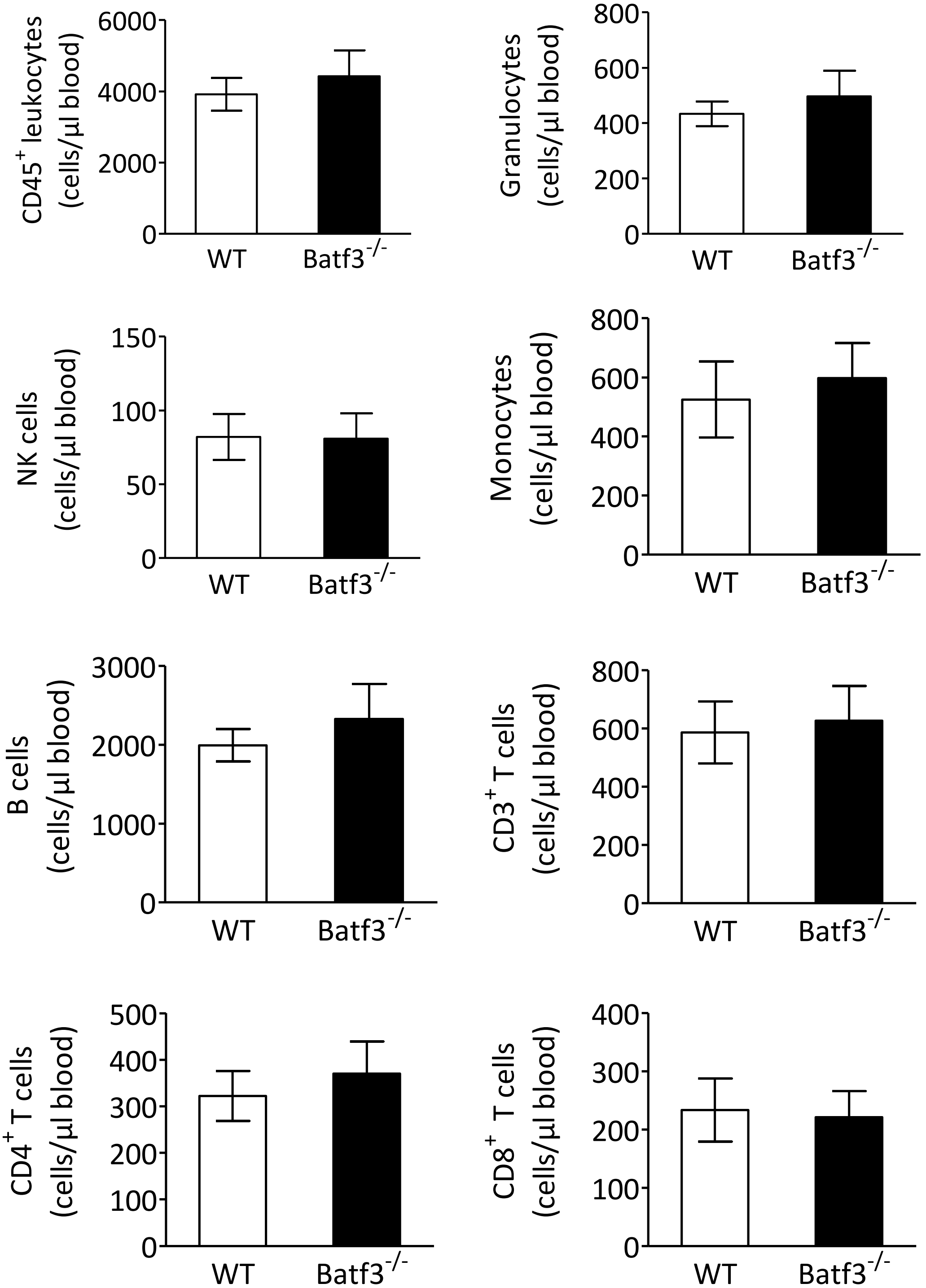

Supplementary Figure S2. Blood leukocyte patterns in wt and *batf3*$^{-/-}$ transplanted *ldlr*$^{-/-}$ mice after 10 weeks of WTD

TruCount tubes and flow cytometry were used to determine exact cell numbers of leukocytes ($CD45^{+}$), granulocytes ($CD11b^{high}$ $Ly6G^{+}$), NK cells ($CD3^{-}$ $NK1.1^{+}$), monocytes ($CD11b^{high}$ $Ly6G^{-}$), B cells ($B220^{+}$), T cells ($CD3^{+}$ $NK1.1^{-}$), $CD4^{+}$ T cells ($CD3^{+}$ $NK1.1^{-}$ $CD4^{+}$) and $CD8^{+}$ T cells ($CD3^{+}$ $NK1.1^{-}$ $CD8^{+}$) in wt and *batf3*$^{-/-}$ transplanted *ldlr*$^{-/-}$ mice after 10 weeks of WTD (n=8). Graphs show number of cells per microliter blood, and depict mean ± SEM.

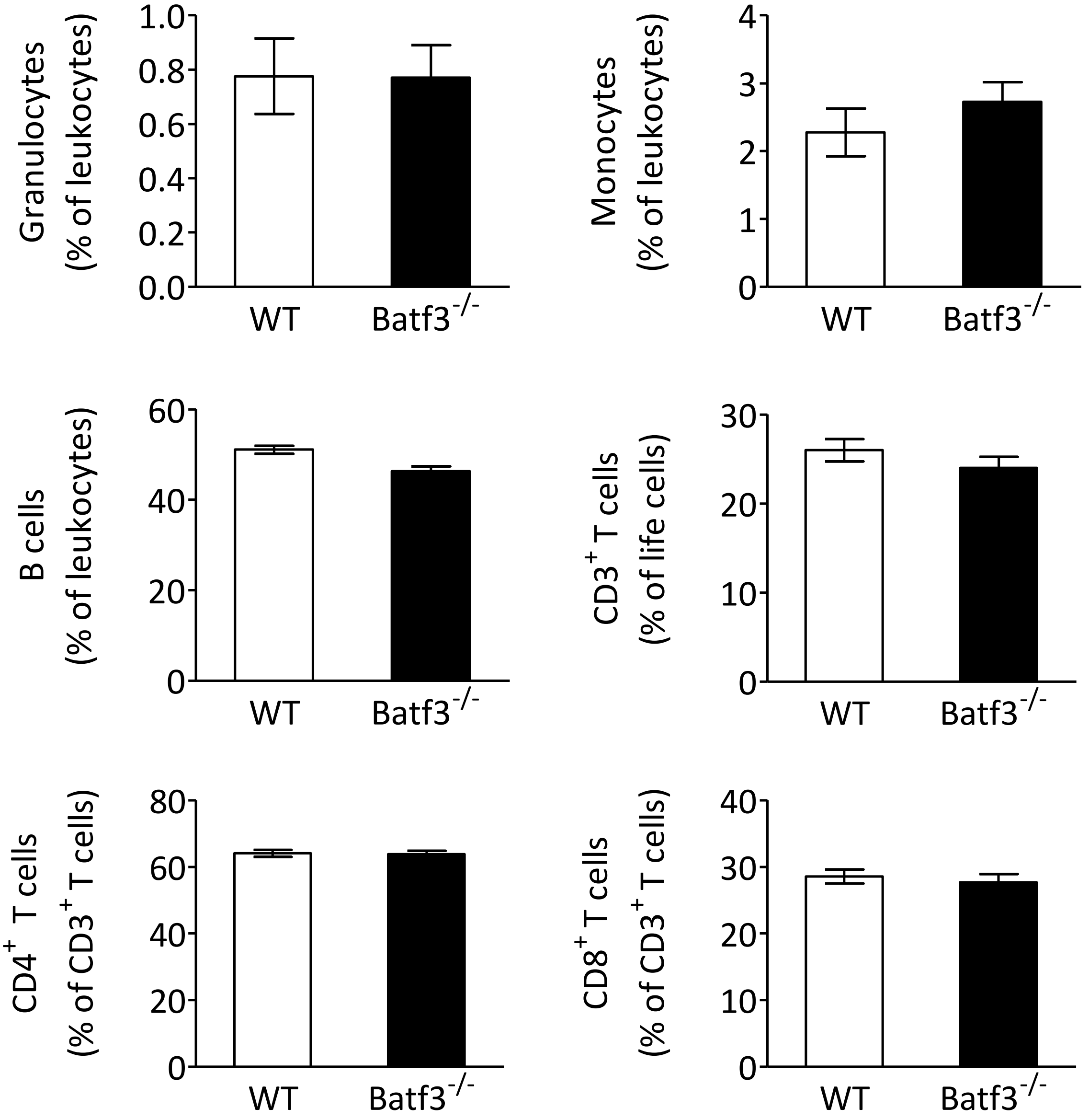

Supplementary Figure S3. Spleen leukocyte subset counts in wt and *batf3*$^{-/-}$ transplanted *ldlr*$^{-/-}$ mice after 10 weeks of WTD

Splenic cell populations were determined by flow cytometry in wt and *batf3*$^{-/-}$ transplanted *ldlr*$^{-/-}$ mice after 10 weeks of WTD (n=8): granulocytes (CD11b^{high} Ly6G^{+}), monocytes (CD11b^{high} Ly6G^{-}), Bcells (B220^{+}) and Tcells (CD3^{+} NK1.1^{-}) are shown as percentage of leukocytes (CD45^{+}), CD4^{+} Tcells (CD3^{+} NK1.1^{-} CD4^{+}) and CD8^{+} Tcells (CD3^{+} NK1.1^{-} CD8^{+}) as percentage of total Tcells. Graphs depict mean $\pm$ SEM.

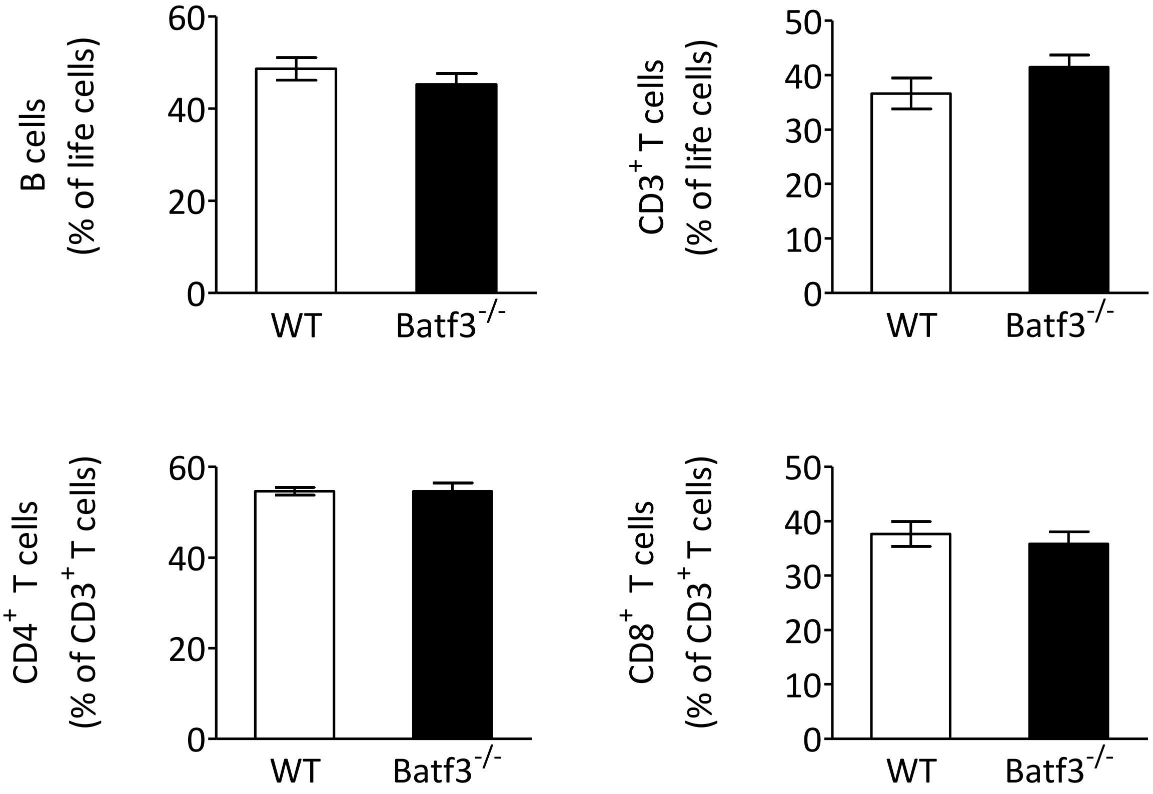

Supplementary Figure S4. Lymph node leukocyte subset distribution in wt and *batf3*$^{-/-}$ transplanted *ldlr*$^{-/-}$ mice after 10 weeks of WTD

Lymphocyte cell populations were determined in a mix of peripheral lymph nodes (axillary, mandibular, mesenteric) by flow cytometry in wt and *batf3*$^{-/-}$ transplanted *ldlr*$^{-/-}$ mice after 10 weeks of WTD (n=8): Bcells (B220^{+}) and Tcells (CD3^{+} NK1.1^{-}) are shown as percentage of life cells. CD4^{+} Tcells (CD3^{+} NK1.1^{-} CD4^{+}) and CD8^{+} Tcells (CD3^{+} NK1.1^{-} CD8^{+}) as percentage of total Tcells. Graphs depict mean $\pm$ SEM.

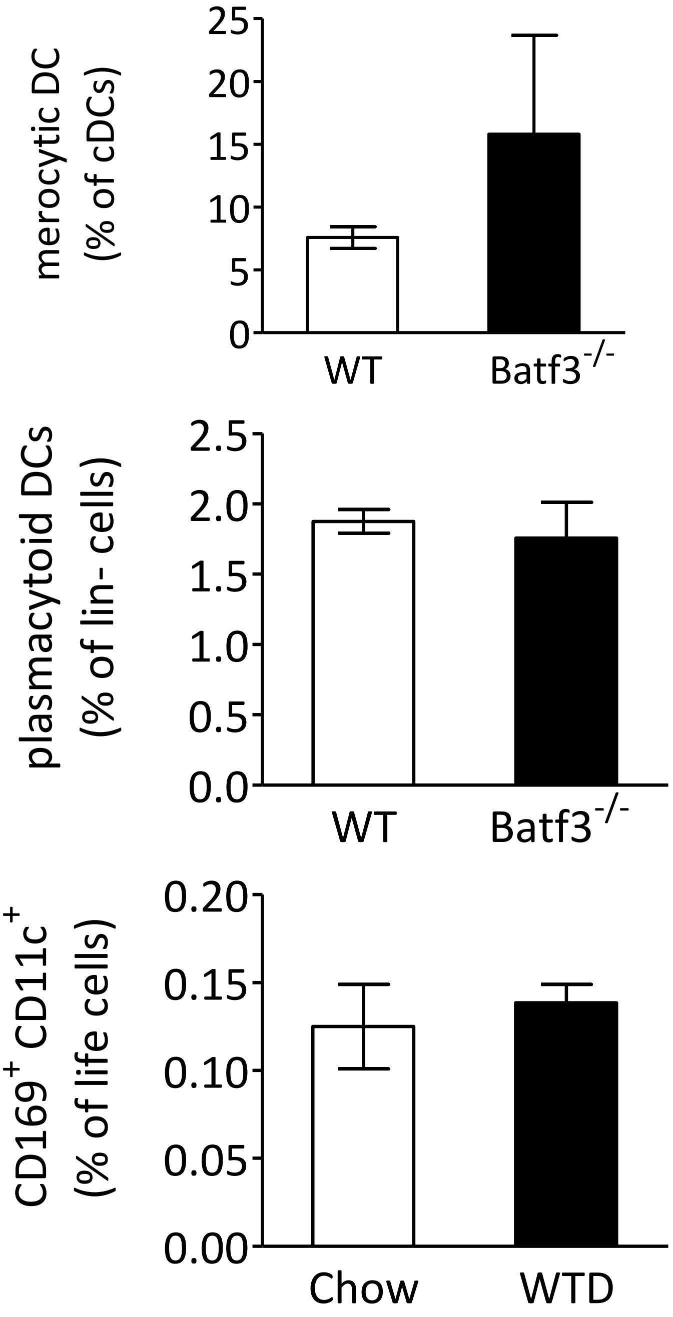

Supplementary Figure S5. Prevalence of other cross-presenting populations in *batf3*$^{-/-}$ chimeric and wt *ldlr*$^{-/-}$ mice

Splenocytes were analyzed by flow cytometry. mDCs as percentage of Lin^{-} CD11c^{high} MHCII^{high} cDCs, pDCs as percentage of Lin^{-} cells. CD169^{+}CD11c^{+} cross-presenting macrophages were compared in mice on chow and after being fed 3 weeks WTD. Data is presented as mean ± SEM.

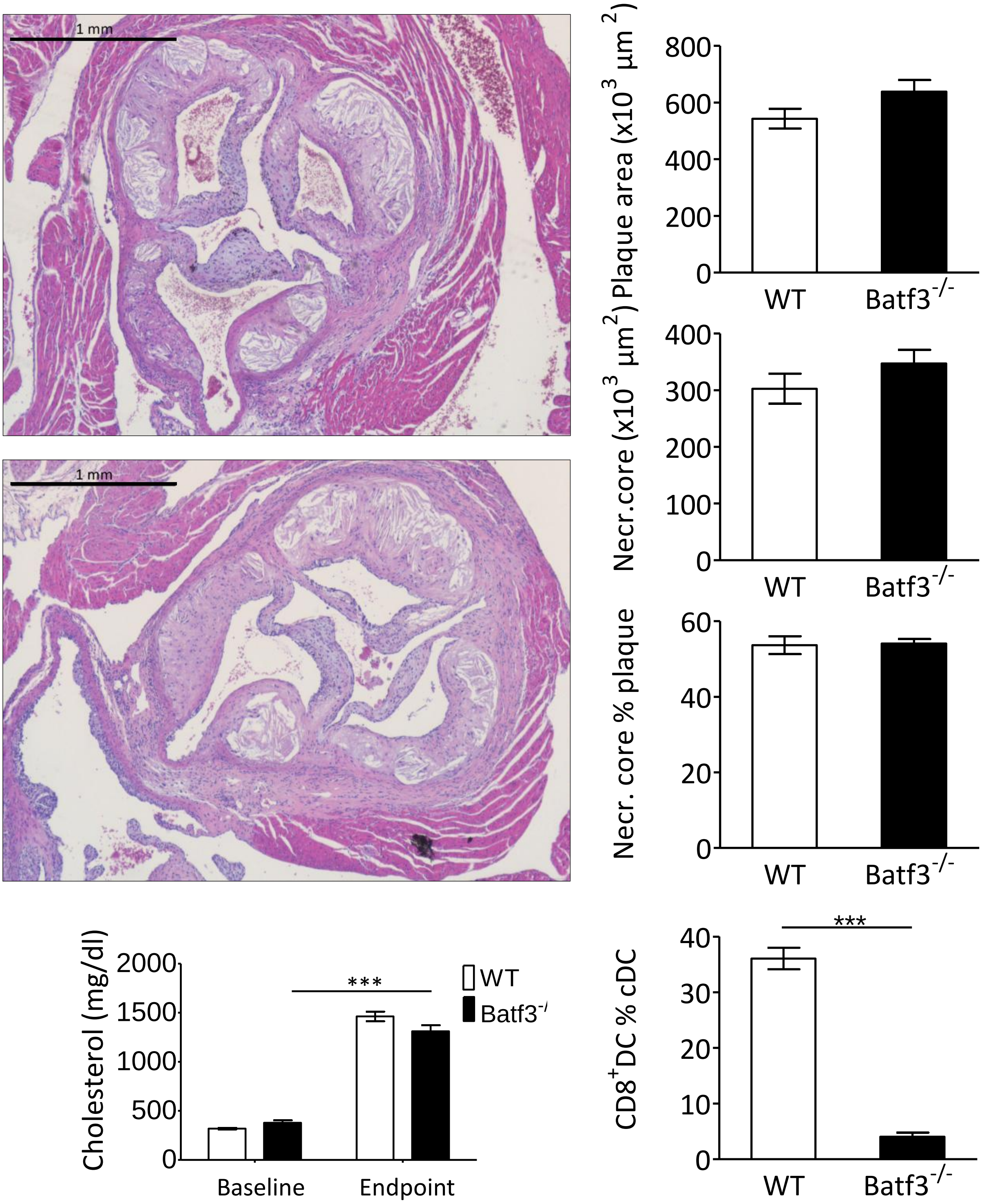

Supplementary Figure S6. Plaque analysis, plasma cholesterol and CD8α⁺ DC depletion in wt and *batf3*$^{-/-}$ transplanted *ldlr*$^{-/-}$ mice of the Cincinnati study

Representative H&E stainings and measurements of plaque area, necrotic core size and percentage necrotic core relative to plaque area are shown for aortic root plaques of wt (n=15) and *batf3*$^{-/-}$ (n=15) transplanted *ldlr*$^{-/-}$ mice. Cholesterol quantity in serum before start of Western Type Diet (baseline) and at sacrifice (endpoint) are shown for wt and *batf3*$^{-/-}$ chimeras. Flow cytometry of splenocytes showing CD8α^{+} DCs as percentage of cDCs (Lin^{-} CD11c^{high} MHCII^{high}). Data is presented as mean SEM, ***: p<0.001.

Supplemental Tables

Supplementary Table I: Primers for cross-presentation markers used in real-time PCR

Gene	NCBI Refseq ID	Forward primer sequence	Reverse primer sequence
**TAP1**	NM_000593.5	5'-gcaagaaataaagacactcaacca-3'	5'-cccactttcagcagcatacc-3'
**ADFP**	NM_001122.3	5'-tcagctccattctactgttcacc-3'	5'-cctgaattttctgattggcact-3'
**BDCA3**	NM_000361.2	5'-aattgggagcttgggaatg-3'	5'-tgaggacctgattaaggctagg-3'
**IRF8**	NM_002163.2	5'-gaggtggtccaggtcttcg-3'	5'-cggccctggctgttatag-3'
**Rab11b**	NM_004218.3	5'-gcattcaagaacatcctcacag-3'	5'-tgatgtccaccacgttgttc-3'
**Necl2**	NM_014333.3	5'-gagttaacatgtgaagccatcg-3'	5'-cgactctcacccaagttacca-3'
**Batf3**	NM_018664.2	5'-cagcgtcctgcagaggag-3'	5'-cttcggaccttcctgtcatc-3'
**β-actin**	NM_001101.3	5'-tcacccacatgtgcccatctacga-3'	5'-cagcggaaccgctcattgccaatgg-3'
