# Supplementary material for: Author Correction: Ablation of CD8α+ dendritic cell mediated cross-presentation does not impact atherosclerosis in hyperlipidemic mice
Source: Sci Rep. 2020 May 26;10:8747. doi: 10.1038/s41598-020-65653-2 (PMC7250898; doi:10.1038/s41598-020-65653-2)
Supplement: Supplementary file 1 — Supplementary Information. [file 41598_2020_65653_MOESM1_ESM.pdf]

Ablation of CD8α+ dendritic cell mediated cross-presentation does not impact atherosclerosis in hyperlipidemic mice

Bart Legein[1], Edith Janssen[2], Thomas Theelen[1], Marion Gijbels[1,4], Joep Walraven[1], Jared Klarquist[2], Cassandra Hennies[2], Kristiaan Wouters[3], Tom T.P. Seijkens[4], Erwin Wijnands[1], Judith Sluimer[1], Esther Lutgens[4,5], Martin Zenke[6], Kai Hildner[7], Erik A.L. Biessen[1], *Lieve Temmerman[1]

[1]Experimental Vascular Pathology, Cardiovascular Research Institute Maastricht (CARIM), University of Maastricht, The Netherlands

[2]Division of Immunobiology, Cincinnati Children's Hospital Research Foundation, and the University of Cincinnati College of Medicine, Cincinnati, OH, United States of America

[3]Department of Internal Medicine, Cardiovascular Research Institute Maastricht (CARIM), University of Maastricht, The Netherlands

[4]Experimental Vascular Biology, Dept. of Medical Biochemistry, Academic Medical Center (AMC), University of Amsterdam, Amsterdam, The Netherlands

[5]Institute for Cardiovascular Prevention (IPEK), Ludwig Maximilians University (LMU), Munich, Germany

[6]Institute for Biomedical Engineering, Dept. of Cell Biology, RWTH Aachen University Medical School, Aachen, Germany

[7]Medical Immunology, Universitatsklinikum Erlangen, Erlangen, Germany

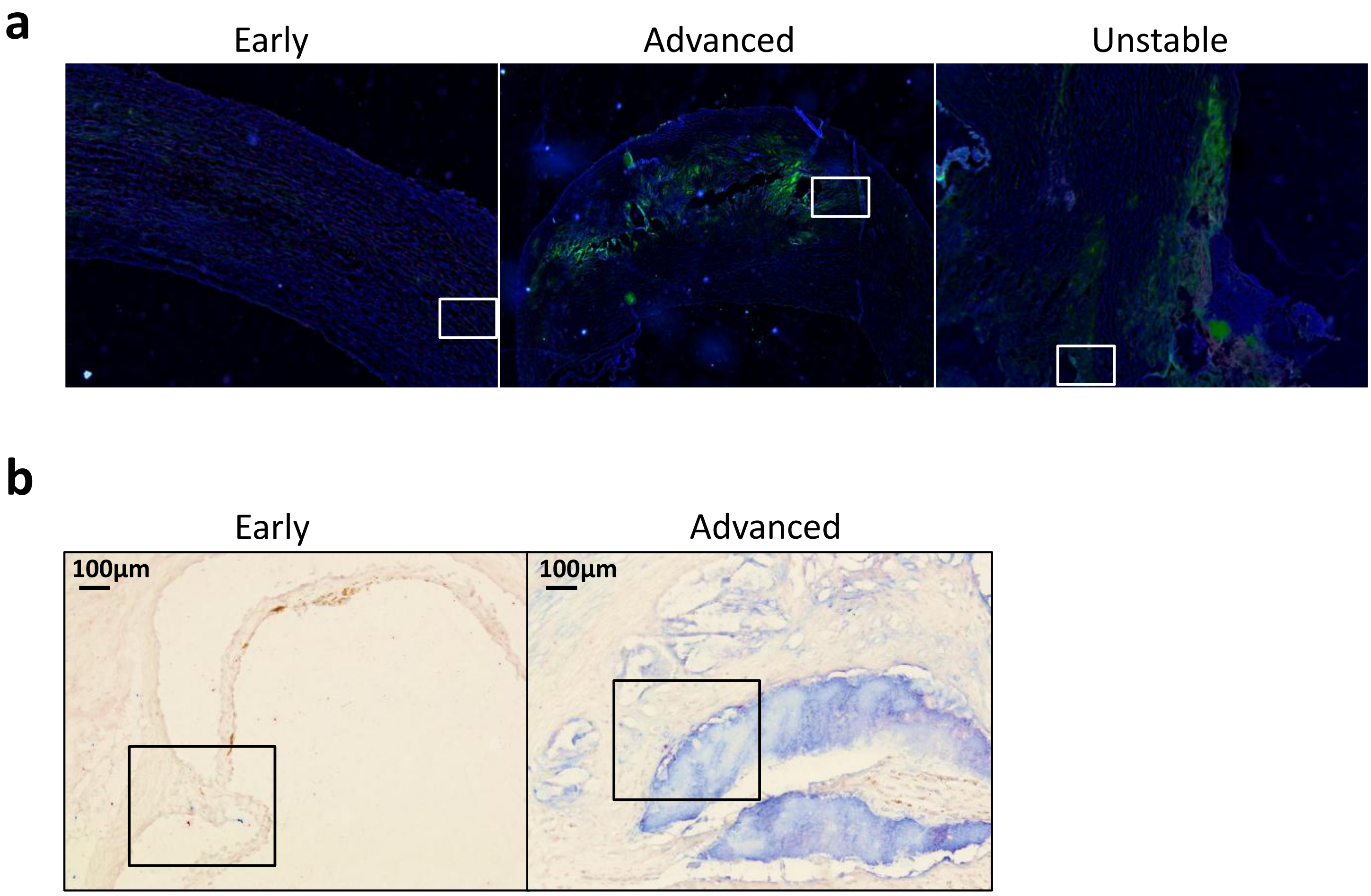

Supplementary Figure S1. Overview images of human and mouse plaque immunohistochemistry

(**a**) Representative images of frozen human carotid plaque sections (n=8-10) doublestained with antibodies against XCR1 (green) and CD11c (red) to identify cross-presenting DCs. Images were acquired with the Nuance Spectral Imaging System and colocalization was analyzed. Background structure of the tissue is indicated in blue. White rectangles indicate regions used in Figure 1b. (**b**) Representative images of frozen mouse aortic root sections doublestained with antibodies against CD8α (red) and CD11c (blue) to identify cross-presenting DCs. Nuclei were lightly counterstained with MethylGreen. Black rectangles indicate regions used in Figure 1d.

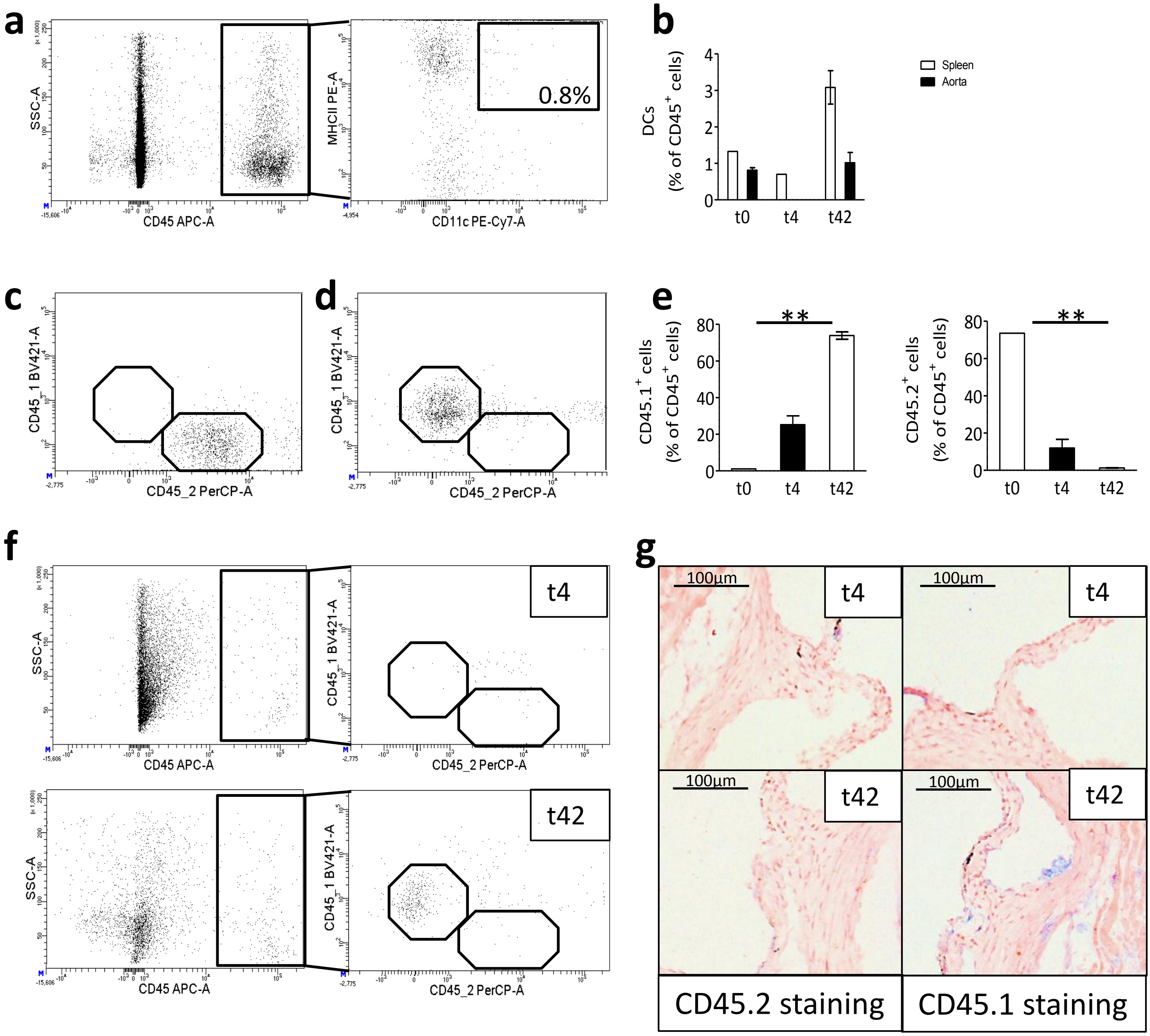

Supplementary Figure S2. CD45.2 immune cells in the vessel wall are replaced by CD45.1 donor cells after bone marrow transplantation

(**a**) Flow cytometry of mouse aorta showing CD45^{+} gate and DC subgate (CD11c^{high}, MHCII^{high}). (**b**) Using gating strategy shown in (a), DC populations in mouse spleen as well as aorta were determined by flow cytometry. t0: control CD45.2^{+} *ldlr*$^{-/-}$ mouse (n=5), t4: CD45.2^{+} *ldlr*$^{-/-}$ mouse 4 days after total body irradiation and transplantation with CD45.1^{+} bone marrow (n=4), t42: CD45.2^{+} *ldlr*$^{-/-}$ mouse 6 weeks after total body irradiation and transplantation with CD45.1^{+} bone marrow (n=8). Data is shown as mean $\pm$ SEM. **: p<0.01. (**c**) CD45.1/CD45.2 flow cytometry plot of the CD45^{+} population in aorta of an acceptor CD45.2^{+} *ldlr*$^{-/-}$ mouse. (**d**) CD45.1/CD45.2 flow cytometry plot of the CD45^{+} population in aorta of a donor CD45.1^{+} *ldlr*$^{-/-}$ mouse. (**e**) Aortic CD45.1^{+} donor-descendant cells (left panel) and CD45.2^{+} acceptor-descendant cells (right panel) were quantified by flow cytometry 4 days (n=4) and 6 weeks after bone marrow transplantation (n=8). (**f**) Representative flow cytometry plots of aortic CD45^{+} cells in CD45.1-transplanted CD45.2^{+} *ldlr*$^{-/-}$ mice 4 days and 6 weeks after transplantation. (**g**) Immunohistochemical staining of CD45.1 (blue) or CD45.2 (blue) positive cells in frozen aortic root sections of CD45.1-transplanted CD45.2^{+} *ldlr*$^{-/-}$ mice 4 days and 6 weeks after transplantation (n=4). Counterstaining: Nuclear Fast Red.

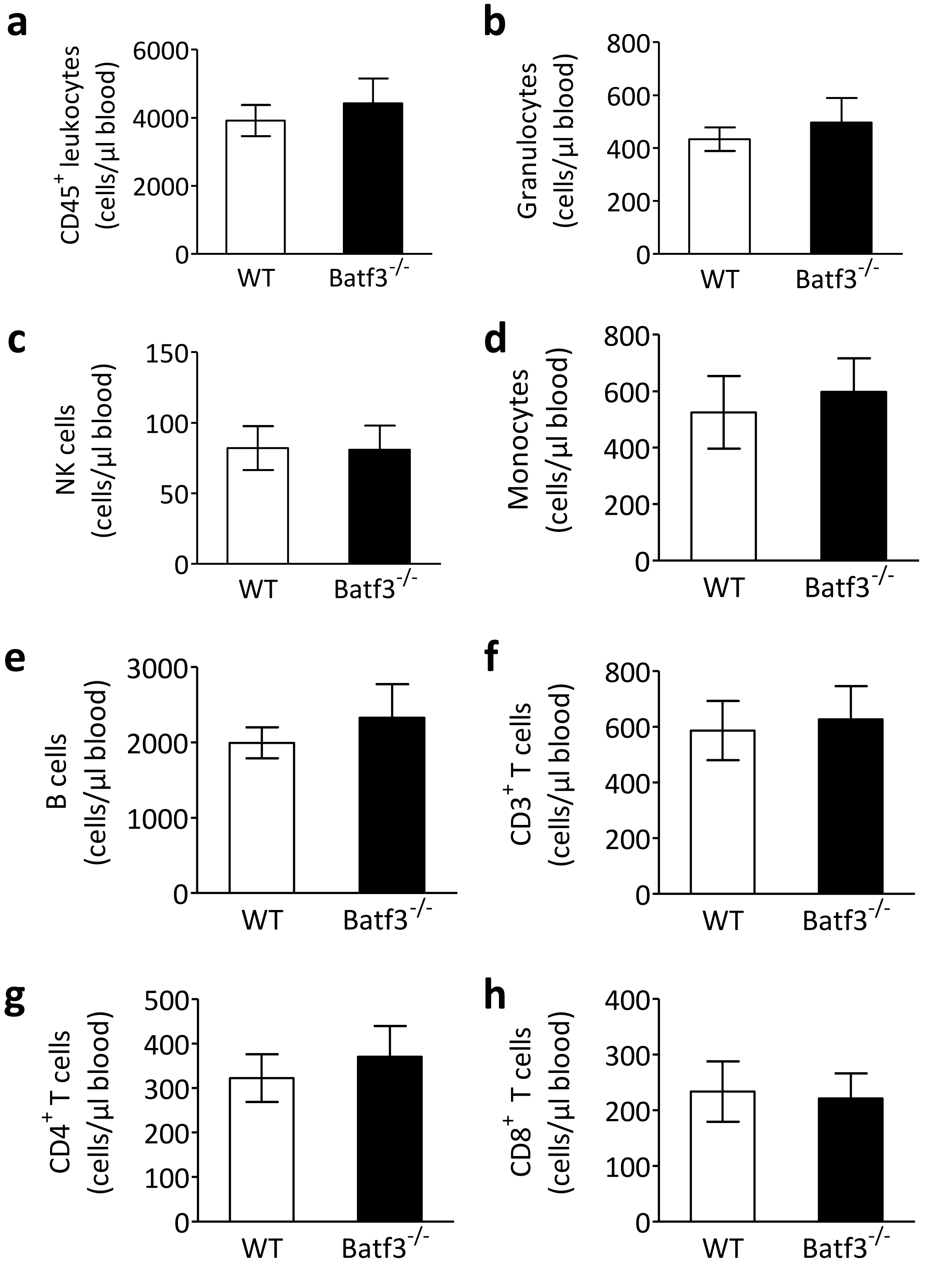

Supplementary Figure S3. Blood leukocyte patterns in wt and *batf3*$^{-/-}$ transplanted *ldlr*$^{-/-}$ mice after 10 weeks of WTD

TruCount tubes and flow cytometry were used to determine exact cell numbers of (**a**) leukocytes ($CD45^+$), (**b**) granulocytes ($CD11b^{high}$ $Ly6G^+$), (**c**) NK cells ($CD3^-$ $NK1.1^+$), (**d**) monocytes ($CD11b^{high}$ $Ly6G^-$), (**e**) B cells ($B220^+$), (**f**) T cells ($CD3^+$ $NK1.1^-$), (**g**) $CD4^+$ T cells ($CD3^+$ $NK1.1^-$ $CD4^+$) and (**h**) $CD8^+$ T cells ($CD3^+$ $NK1.1^-$ $CD8^+$) in wt and *batf3*$^{-/-}$ transplanted *ldlr*$^{-/-}$ mice after 10 weeks of WTD (n=8). Graphs show number of cells per microliter blood, and depict mean ± SEM.

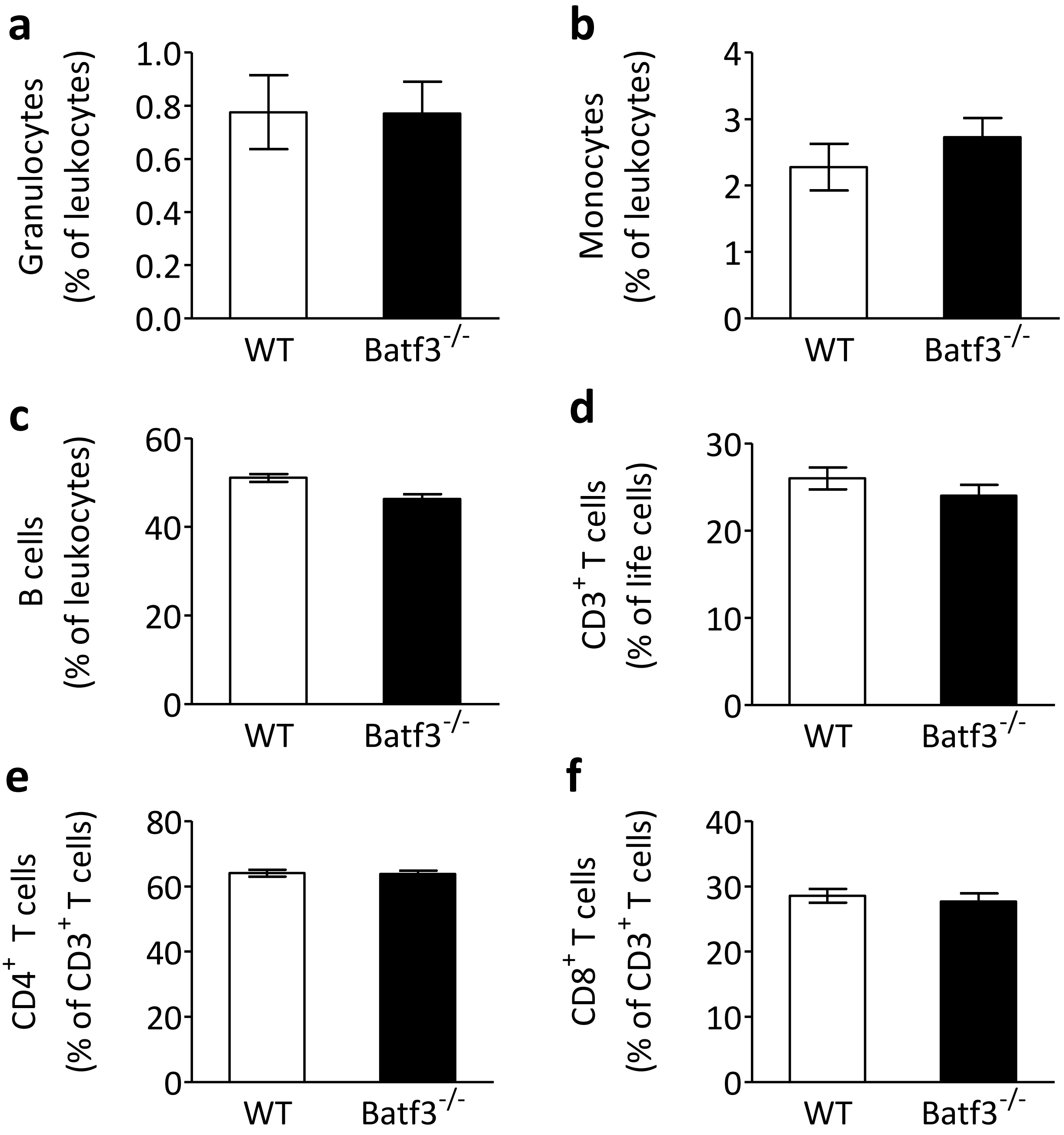

Supplementary Figure S4. Spleen leukocyte subset counts in wt and *batf3*$^{-/-}$ transplanted *ldlr*$^{-/-}$ mice after 10 weeks of WTD

Splenic cell populations were determined by flow cytometry in wt and *batf3*$^{-/-}$ transplanted *ldlr*$^{-/-}$ mice after 10 weeks of WTD (n=8): (**a**) granulocytes (CD11b^{high} Ly6G^{+}), (**b**) monocytes (CD11b^{high} Ly6G^{-}), (**c**) Bcells (B220^{+}) and (**d**) Tcells (CD3^{+} NK1.1^{-}) are shown as percentage of leukocytes (CD45^{+}), (**e**) CD4^{+} Tcells (CD3^{+} NK1.1^{-} CD4^{+}) and (**f**) CD8^{+} Tcells (CD3^{+} NK1.1^{-} CD8^{+}) as percentage of total Tcells. Graphs depict mean $\pm$ SEM.

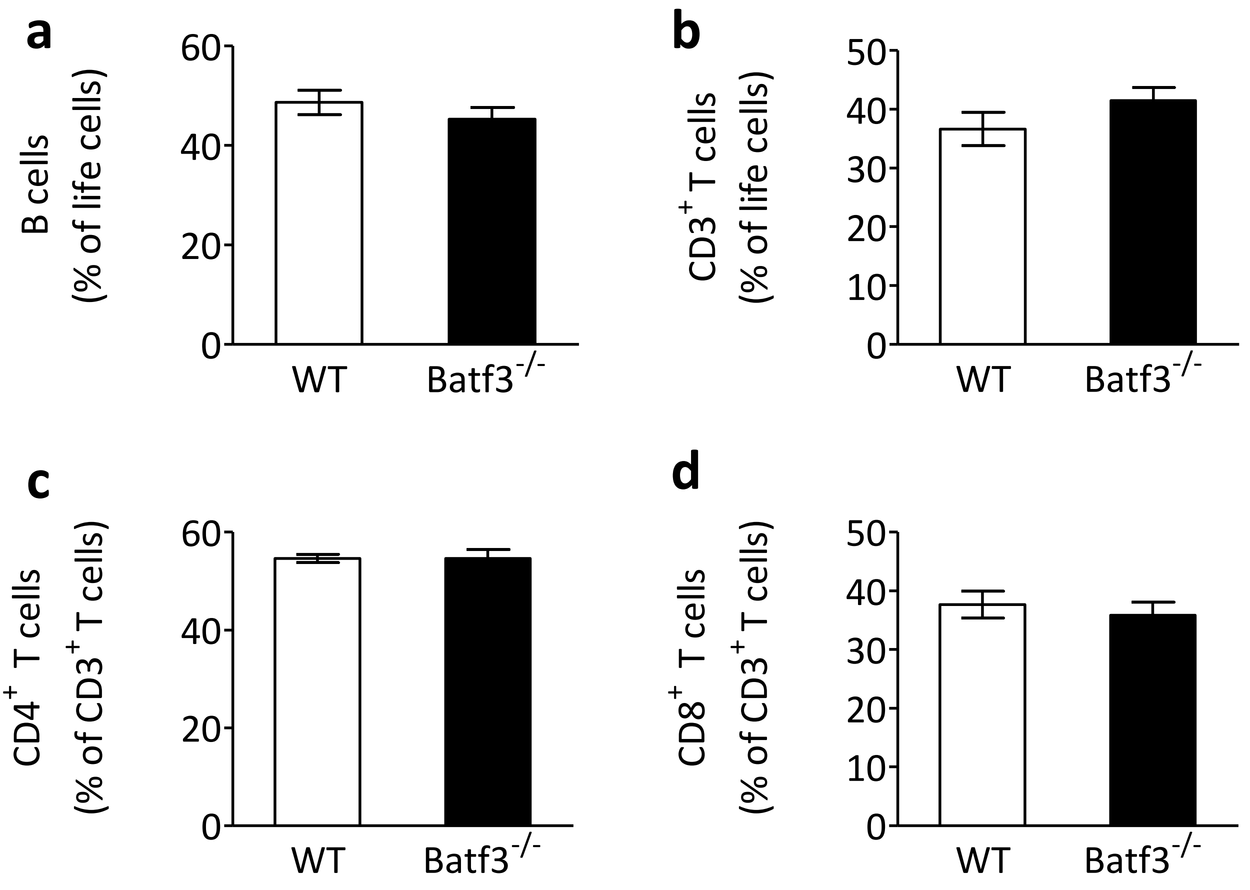

Supplementary Figure S5. Lymph node leukocyte subset distribution in wt and *batf3*$^{-/-}$ transplanted *ldlr*$^{-/-}$ mice after 10 weeks of WTD

Lymphocyte cell populations were determined in a mix of peripheral lymph nodes (axillary, mandibular, mesenteric) by flow cytometry in wt and *batf3*$^{-/-}$ transplanted *ldlr*$^{-/-}$ mice after 10 weeks of WTD (n=8): (**a**) Bcells (B220$^+$) and (**b**) Tcells (CD3$^+$ NK1.1$^-$) are shown as percentage of life cells. (**c**) CD4$^+$ Tcells (CD3$^+$ NK1.1$^-$ CD4$^+$) and (**d**) CD8$^+$ Tcells (CD3$^+$ NK1.1$^-$ CD8$^+$) as percentage of total Tcells. Graphs depict mean $\pm$ SEM.

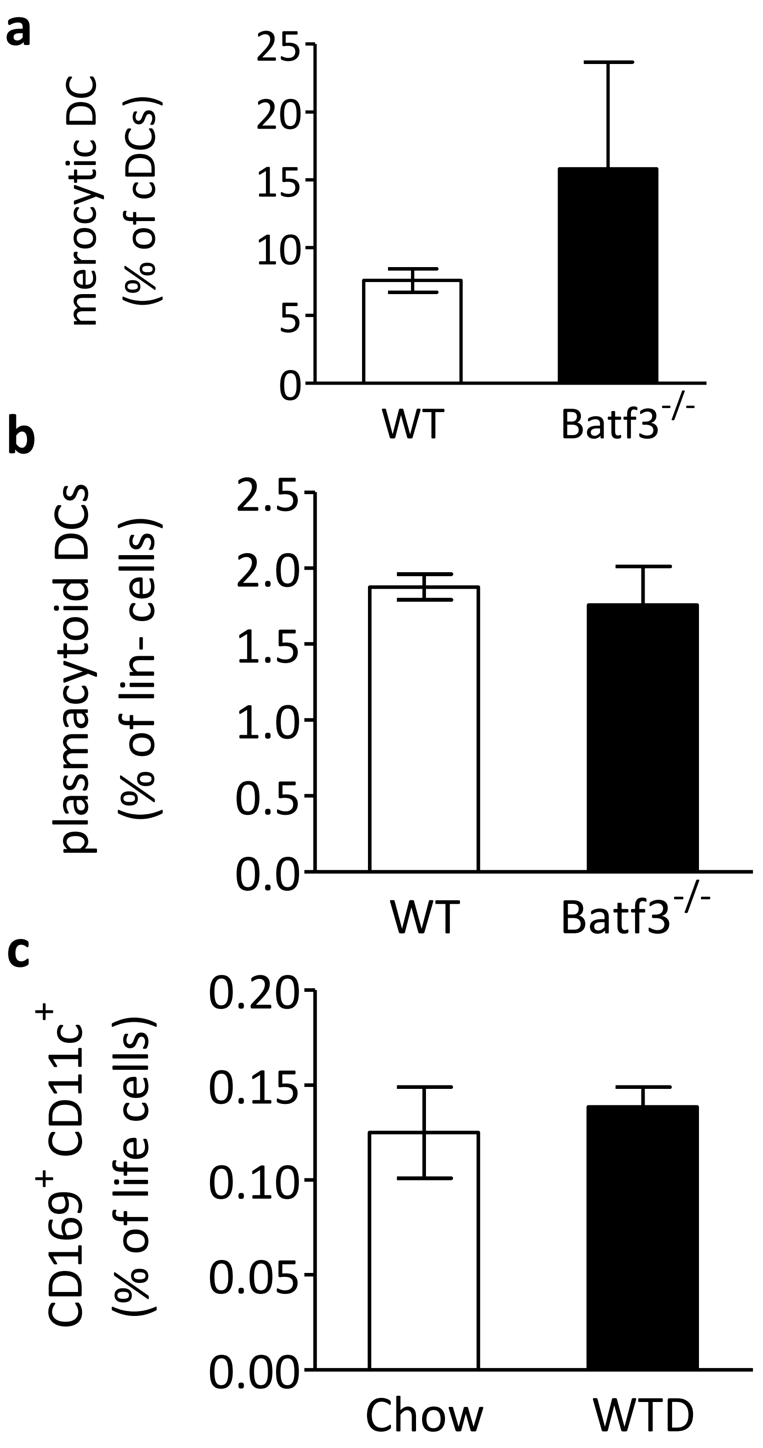

Supplementary Figure S6. Prevalence of other cross-presenting populations in *batf3*$^{-/-}$ chimeric and wt *ldlr*$^{-/-}$ mice

(**a**, **b**) Splenocytes were analyzed by flow cytometry in *batf3*$^{-/-}$ chimeric and wt *ldlr*$^{-/-}$ mice after 10 weeks of WTD. Graphs depict (**a**) mDCs as percentage of Lin^{-} CD11c^{high} MHCII^{high} cDCs, (**b**) pDCs as percentage of Lin^{-} cells. (**c**) CD169^{+}CD11c^{+} cross-presenting macrophages were compared in mice on chow and after being fed 3 weeks WTD. Data is presented as mean ± SEM.

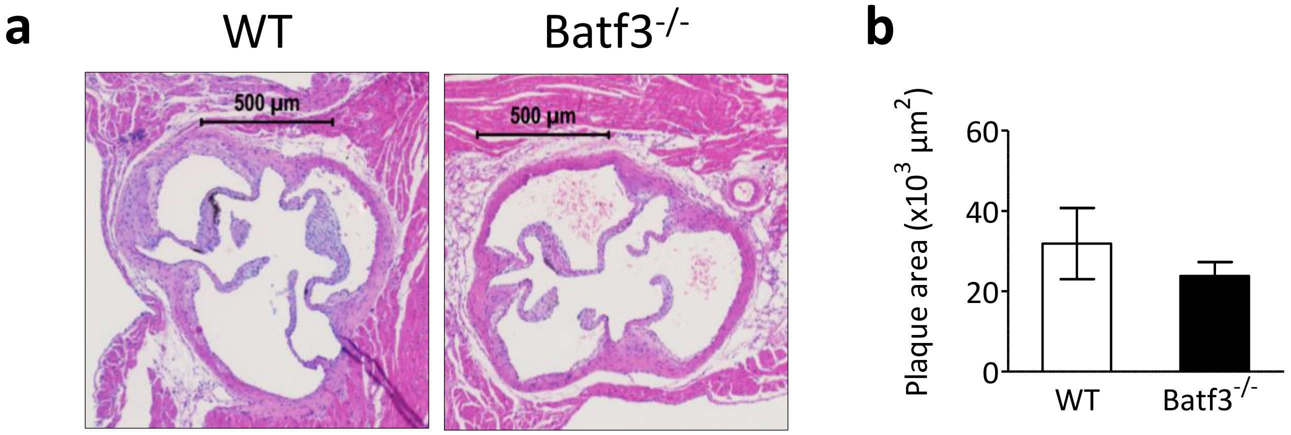

Supplementary Figure S7. Plaque analysis in wt and *batf3*$^{-/-}$ transplanted *ldlr*$^{-/-}$ mice on normal chow diet

(**a**) Representative H&E stainings and (**b**) measurements of plaque area, are shown for aortic root plaques of wt (n=3) and *batf3*$^{-/-}$ (n=4) transplanted *ldlr*$^{-/-}$ mice. Data is presented as mean ± SEM.

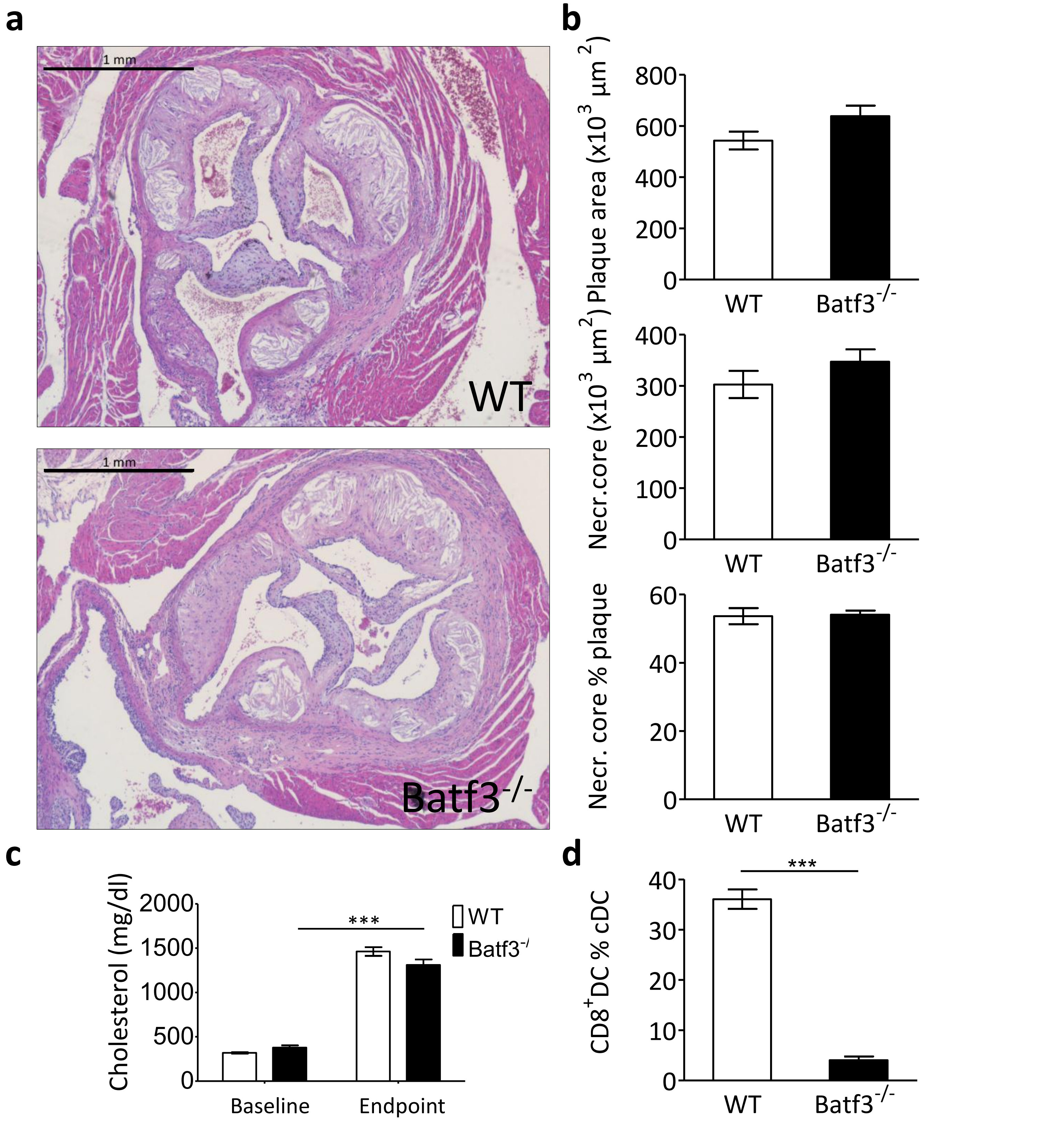

Supplementary Figure S8. Plaque analysis, plasma cholesterol and CD8α⁺ DC depletion in wt and *batf3*$^{-/-}$ transplanted *ldlr*$^{-/-}$ mice of the Cincinnati study

(**a**) Representative H&E stainings and (**b**) measurements of plaque area, necrotic core size and percentage necrotic core relative to plaque area are shown for aortic root plaques of wt (n=15) and *batf3*$^{-/-}$ (n=15) transplanted *ldlr*$^{-/-}$ mice. (**c**) Cholesterol quantity in serum before start of Western Type Diet (baseline) and at sacrifice (endpoint) are shown for wt and *batf3*$^{-/-}$ chimeras. (**d**) Flow cytometry of splenocytes showing CD8α⁺ DCs as percentage of cDCs (Lin^{-} CD11c^{high} MHCII^{high}). Data is presented as mean ± SEM, ***: $p<0.001$.

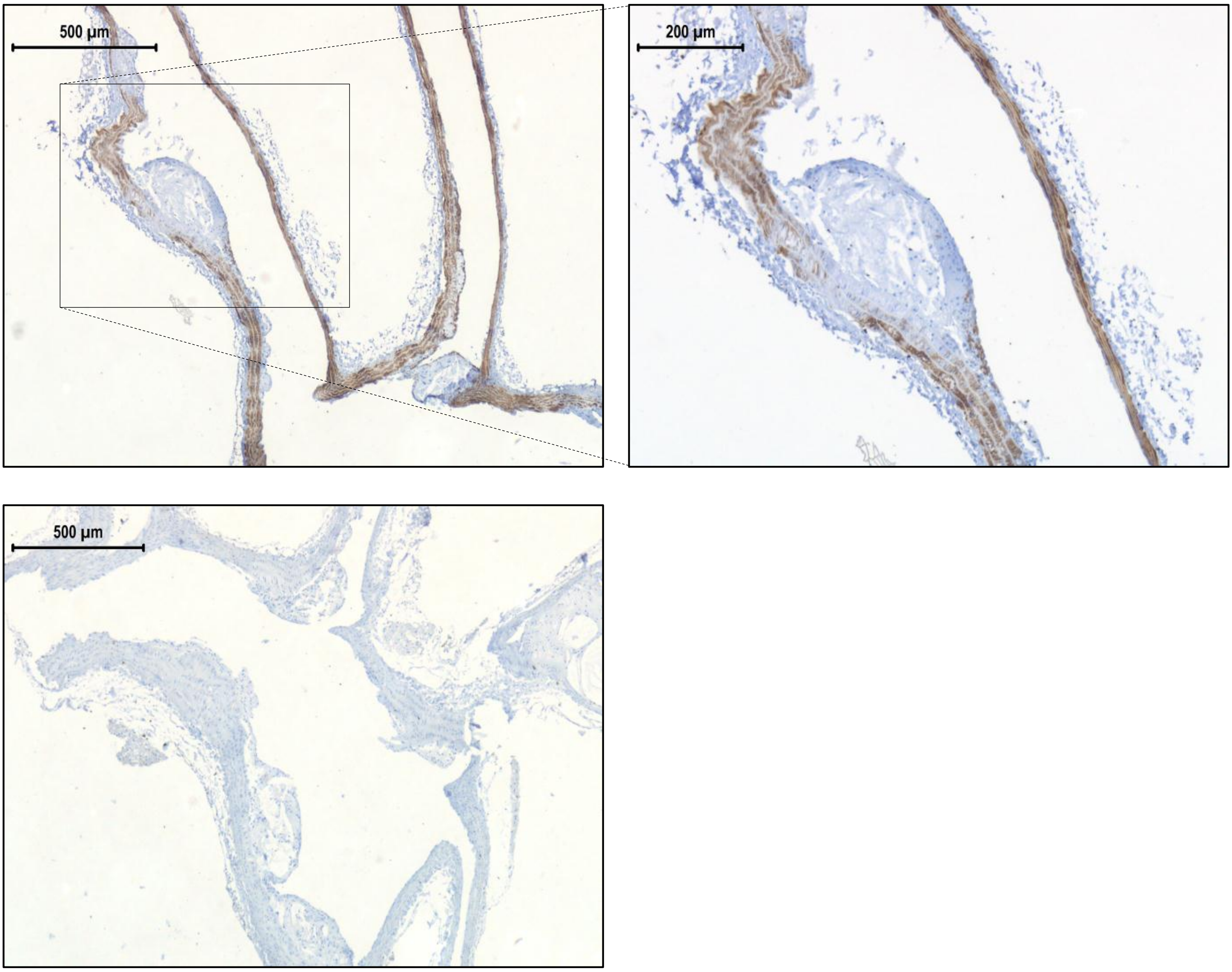

Supplementary Figure S9. Staining control for αSMA immunohistochemistry

Aortic arches of wt and *batf3*$^{-/-}$ transplanted *ldlr*$^{-/-}$ mice were taken along as staining controls for the αSMA immunohistochemistry performed on aortic root sections shown in Figure 6. Top panels: positive control showing clear αSMA staining in the media, lower panel: negative isotope control.

Supplemental Tables

Supplementary Table I: Primers for cross-presentation markers used in real-time PCR

Gene	NCBI Refseq ID	Forward primer sequence	Reverse primer sequence
**hTAP1**	NM_000593.5	5’-gcaagaaataaagacactcaacca-3’	5’-cccactttcagcagcatacc-3'
**hADFP**	NM_001122.3	5’-tcagctccattctactgttcacc-3’	5’-cctgaattttctgattggcact-3’
**hBDCA3**	NM_000361.2	5’-aattgggagcttgggaatg-3’	5’-tgaggacctgattaaggctagg-3’
**hIRF8**	NM_002163.2	5’-gaggtggtccaggtcttcg-3’	5’-cggccctggctgttatag-3’
**hNecl2**	NM_014333.3	5’-gagttaacatgtgaagccatcg-3’	5’-cgactctcacccaagttacca-3’
**hBatf3**	NM_018664.2	5’-cagcgtcctgcagaggag-3’	5’-cttcggaccttcctgtcatc-3’
**hRab11b**	NM_004218.3	5’-gcattcaagaacatcctcacag-3’	5’-tgatgtccaccacgttgttc-3’
**hβ-actin**	NM_001101.3	5’-tcacccacatgtgcccatctacga-3’	5’-cagcggaaccgctcattgccaatgg-3’
**mRab11b**	ENSMUST00000172894.1	5’-ggaggttggtaggatggaca-3’	5’-gcggtttcgatctctgaagt-3’
**mTAP1**	ENSMUST00000171148.1	5’-gggtgaggcccagaagtt-3’	5’-gcagcattcccgagacac-3’
**mXCR1**	NM_011798.4	5’-ctcaacctgtgtctctcagacct-3’	5’-aaccaactccattgtgctga-3’

Supplementary Table II: Characteristics of wt and *batf3*$^{-/-}$ transplanted *ldlr*$^{-/-}$ mice

	BMT	n	Age (wks) at BMT	Body weight at sacrifice (g)	Cholesterol at sacrifice (mg/dl)	Diet period (wks)
**Maastricht Study**	wt	15	23 ± 3 days	23.6 ± 0.9	1301 ± 110.0	10
	batf3$^{-/-}$	12	22 ± 3 days	24.5 ± 1.3	1168 ± 62.3	10
**Cincinnati Study**	wt	10	16 ± 2 days	23.5 ± 0.6	1462 ± 49.1	10
	batf3$^{-/-}$	10	16 ± 2 days	23.8 ± 0.7	1310 ± 62.6	10

Supplemental References

1. Virmani R, Kolodgie FD, Burke AP, Farb A, Schwartz SM. Lessons from sudden coronary death: a comprehensive morphological classification scheme for atherosclerotic lesions. *Arteriosclerosis, thrombosis, and vascular biology.* May 2000;20(5):1262-1275.

2. Sambrook J, Fritsch E, Maniatis T. *Molecular cloning: a laboratory manual.* Vol 2. Cold Spring Harbor, NY: Cold Spring Harbor Laboratory Press; 1989.

3. Lutgens E, Gorelik L, Daemen MJ, de Muinck ED, Grewal IS, Koteliansky VE, Flavell RA. Requirement for CD154 in the progression of atherosclerosis. *Nature medicine.* Nov 1999;5(11):1313-1316.
